# Mitochondrial superoxide–induced mitohormesis is mediated by citrate and cardioprotective

**DOI:** 10.1126/sciadv.aef8132

**Published:** 2026-09-04

**Authors:** Matthew P. Donnelly, Kailash Chandra Mangalhara, Yuening Liu, Kathryn Lande, Gladys R. Rojas, Kym J. Grae, Mack B. Reynolds, Sagnika Ghosh, Neva Olliffe, Pau B. Esparza-Moltó, Melissa A. Johnson, Suzanne Dufresne, Allison Y. Louie, Alexandra G. Moyzis, Deann Guan, Åsa B. Gustafsson, Christina G. Towers, Pallav Kosuri, Christian M. Metallo, Diana C. Hargreaves, Gerald S. Shadel

**Affiliations:** ^1^ Salk Institute for Biological Studies, La Jolla, CA, USA.; ^2^ Medical Scientist Training Program, University of California San Diego School of Medicine, La Jolla, CA, USA.; ^3^ Biomedical Sciences Graduate Program, University of California, San Diego, La Jolla, CA, USA.; ^4^ Sanford Burnham Prebys Medical Discovery Institute, La Jolla, CA, USA.; ^5^ Biological Sciences Graduate Program, University of California San Diego, La Jolla, CA, USA.; ^6^ Skaggs School of Pharmacy and Pharmaceutical Sciences, University of California San Diego, La Jolla, CA, USA.

## Abstract

Mitohormesis, whereby transient mitochondrial stress induces adaptive signaling, promotes organismal resilience and longevity in invertebrates, but how this operates in mammals and the underlying metabolic signals involved remain unclear. Using a mouse model of mitohormesis, we show that transient mitochondrial superoxide stress during embryogenesis reprograms the adult heart to enhance mitochondrial biogenesis and antioxidant capacity. These adaptations confer protection against mitochondrial and oxidative injury in models of doxorubicin-induced cardiotoxicity, preserving mitochondrial content and preventing cardiac dysfunction and remodeling. Using a cell model of superoxide-mediated mitohormesis, we find that inhibition of mitochondrial aconitase promotes citrate export to the cytosol, where its conversion to acetyl–coenzyme A drives histone acetylation and mitohormetic protection from oxidative stress. Preventing mitochondrial citrate export abolishes these adaptations, while *Aco2* silencing or citrate supplementation recapitulates the response. Together, our findings identify mitochondrial citrate as a redox-sensitive second messenger linking mitochondrial superoxide stress to durable epigenetic and mitohormetic remodeling.

## INTRODUCTION

Mitochondria are multifunctional organelles central to cellular and organismal physiology whose composition, morphology, and activities are tailored to specific cell and tissue types (*1*). While well-known for their role in energy metabolism and adenosine 5′-triphosphate (ATP) production via oxidative phosphorylation (OXPHOS), mitochondria are also signaling hubs (*1*, *2*). By sensing diverse physiological and pathophysiological stimuli, mitochondria can coordinate adaptive responses that govern a large range of biological functions (*2*). A common way this occurs is through mitochondria-derived signaling mediators, such as nucleic acids, metabolites, and reactive oxygen species (ROS), which directly or indirectly (i.e., via cytoplasmic sensors) reprogram nuclear gene expression (*3*–*5*). Relevant to the current study, transient exposure to mitochondrial stress can activate signaling responses that confer future stress resistance through a process termed “mitohormesis” (*6*). In invertebrate model systems, including budding yeast (*Saccharomyces cerevisiae*), worms (*Caenorhabditis elegans*), and flies (*Drosophila melanogaster*), mitohormetic perturbations can even extend life span (*7*–*9*).

Mitochondria are a major source of ROS, harboring multiple enzymatic sites that produce superoxide or hydrogen peroxide (*10*). Accordingly, various antioxidant enzymes reside in mitochondria, including superoxide dismutase 1 (SOD1) and SOD2 that detoxify superoxide in the intermembrane space and mitochondrial matrix, respectively (*11*). While once considered purely damaging, mitochondrial ROS are now recognized as essential signaling molecules that mediate physiological and stress responses, including cell differentiation and proliferation, senescence, autophagy, apoptosis, development, and immunity (*12*–*16*). Consistent with this, antioxidants have been shown to prevent the benefits of exercise and can even worsen clinical outcomes, highlighting the importance of regulated ROS signaling (*17*–*20*). In many cases, mitohormetic adaptations, including life-span extension, are abolished by antioxidants, emphasizing the role of ROS in this process (*21*). Furthermore, work in *C. elegans* confirmed that mitochondrial matrix localized superoxide specifically initiates mitohormesis to increase life span (*22*). We previously showed that mitohormesis driven by inhibition of SOD2 expression (and thus increased matrix superoxide) only during mouse embryonic development remodeled adult livers to increase mitochondrial biogenesis and basal antioxidant gene expression (*23*). While this study directly illustrated that superoxide-mediated mitohormesis is conserved in mammals, whether this enhances stress resistance and can decrease disease susceptibility in the adapted animals remained untested. Moreover, how a mitochondrial matrix superoxide signal is relayed to the nucleus to alter gene expression was not defined.

Due to its extraordinary energy demand, the heart relies on mitochondrial OXPHOS for ∼90% of ATP production (*24*). Consequently, mitochondrial dysfunction and oxidative stress are main drivers of cardiovascular disease (*25*, *26*). The chemotherapeutic drug doxorubicin (DOXO) causes a dose-dependent cardiac injury characterized by mitochondria-derived inflammation, impaired mitochondrial biogenesis, and oxidative stress (*27*–*31*). Despite advances in understanding the mitochondrial contribution to DOXO-induced cardiotoxicity (DIC), no effective mitochondrial or antioxidant therapies exist. Unexpectedly, initial strategies aimed at enhancing mitochondrial biogenesis or antioxidant capacity using genetically engineered mice exacerbated multiple models of cardiomyopathy, suggesting that cardiac function requires a redox-optimized ROS balance (*32*–*36*). In this study, we tested whether mitohormesis could reprogram the adult heart through these pathways without inducing cardiac damage and, by doing so, provide protection from DIC in mice. Using the iSOD2 mouse model, we found that exposure to mitochondrial superoxide stress during development reprogrammed the adult heart to increase mitochondrial biogenesis and antioxidant enzyme expression, which conferred protection in acute and chronic models of DIC. Furthermore, we showed that elevated mitochondrial matrix superoxide signals to the nucleus by inactivating the tricarboxylic acid (TCA) cycle enzyme aconitase, thereby promoting the efflux of its substrate citrate from mitochondria. Once in the cytosol, citrate is converted to acetyl–coenzyme A (CoA), a cofactor needed for histone acetylation. Together, these results provide insight into the cardioprotective potential of mitohormesis and possible therapeutic approaches for DIC, as well as identify citrate as a redox-sensitive second messenger of mitochondrial retrograde and mitohormetic signaling.

## RESULTS

### Transient mitochondrial superoxide stress during development results in adaptive mitochondrial and antioxidant responses in the adult heart

**Fig. 1.**
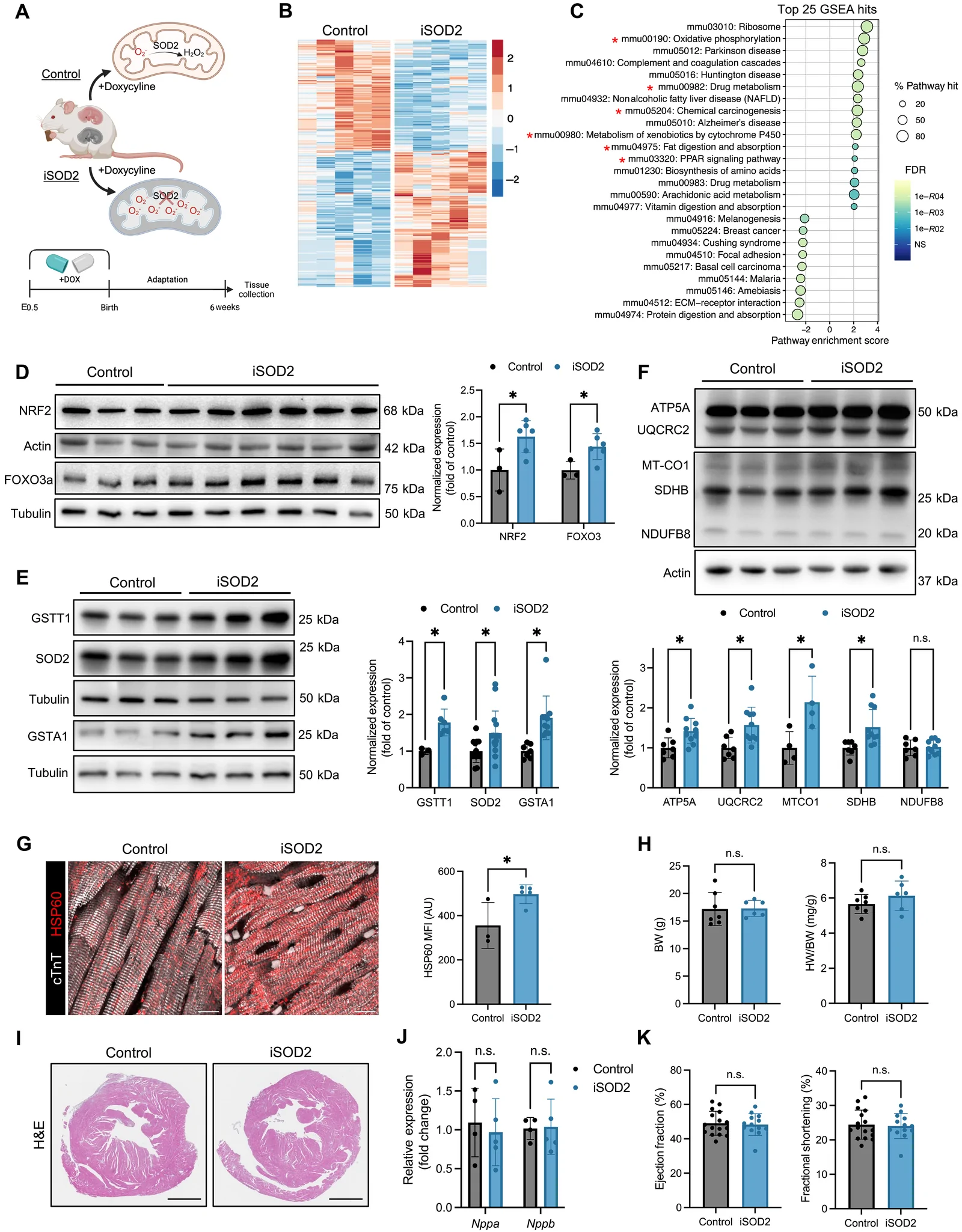
Transient increase in mitochondrial matrix superoxide during embryogenesis induces sustained mitohormetic adaptations in the adult heart. (**A**) Schematic depicting experimental design for inducible knockdown of superoxide dismutase 2 (*Sod2*) in iSOD2 mice during embryogenesis and postnatal adaptation. DOX is doxycycline. (**B**) Heatmap of significant differentially expressed genes between control and iSOD2 mice determined from RNA-seq of male and female 6-week-old heart tissue. Color depicts *z*-score. *P* adj. < 0.05 and log_2_FC > 0.25 (*n* = 5 mice per condition). (**C**) Top 25 enriched pathways in iSOD2 versus control mice determined with gene set enrichment analysis (GSEA) of RNA-seq dataset. FDR, false discovery rate, * depicts proposed mitohormetic pathways (*n* = 5 mice per condition). PPAR, peroxisome proliferator–activated receptor. (**D**) Western blots of heart lysates from 6-week-old control versus iSOD2 mice for transcriptional regulators of mitohormesis (*n* = 3 controls and *n* = 6 iSOD2 mice). (**E** and **F**) Western blot analysis confirming antioxidant and mitochondrial biogenesis transcriptional responses. (E) NRF2 and FOXO3 antioxidant targets and (F) OXPHOS proteins from heart lysates (*n* = 3 to 13 controls and *n* = 6 to 15 iSOD2 mice). (**G**) Representative immunofluorescence for cardiomyocyte (cTnT^+^)–specific mitochondrial signal in control and iSOD2 mice (HSP60 depicts mitochondria, *n* = 3 control and *n* = 5 iSOD2 mice). Scale bars, 10 μm. (**H**) Body weight (BW) and heart weight–to–body weight ratio (HW/BW) of control and iSOD2 mice at 6 weeks (*n* = 7 control and *n* = 6 iSOD2 mice). (**I**) Representative hematoxylin and eosin (H&E) staining of 3-month-old heart cross sections showing no cardiac remodeling (*n* = 5 mice per condition). Scale bars, 1 mm. (**J**) Reverse transcription quantitative polymerase chain reaction (RT-qPCR) analysis of natriuretic peptides *Nppa* and *Nppb* as markers of cardiac stress in 3-month-old hearts (*n* = 5 controls and *n* = 6 iSOD2 mice). (**K**) Cardiac function measured by ejection fraction and fractional shortening in percentage using echocardiography in 3-month-old mice illustrating no cardiac dysfunction in iSOD2 mice (*n* = 16 control and *n* = 13 iSOD2 mice). Significance determined using unpaired *t* test. *P < 0.05; n.s., not significant. Histograms represent means ± SEM. Schematic created in BioRender. Donnelly, M. (2026) https://BioRender.com/t9jne5s.

Using the inducible and reversible *Sod2* knockdown mouse model (iSOD2 mice) that we previously developed (*23*), we asked whether superoxide-mediated mitohormetic adaptations occur within the physiological window required for cardiac function in adult hearts. In the iSOD2 model, doxycycline administration triggers expression of a short hairpin RNA (shRNA) targeting *Sod2*. Crossing a wild-type C57BL/6J female with a male homozygous for the doxycycline-inducible reverse tetracycline-controlled transactivator (rtTA) system and heterozygous for the *Sod2* shRNA generated two experimental groups: (i) intrauterine littermate controls exposed to doxycycline but lacking the shRNA and (ii) iSOD2 mice harboring one copy of the *Sod2* shRNA that experience SOD2 loss and thus increased superoxide accumulation. Doxycycline chow was provided to pregnant dams from embryonic day 0.5 (E0.5) until birth to induce embryonic mitochondrial oxidative stress in iSOD2 mice via *Sod2* knockdown (Fig. 1A). Embryonic heart tissue was collected at E17.5 and confirmed by α-actinin expression with Western blot analysis (fig. S1A). Embryonic iSOD2 hearts exhibited substantial loss of SOD2 protein and elevated expression of the antioxidant transcriptional regulator NRF2 compared with littermate controls, confirming that oxidative stress was induced by SOD2 loss (fig. S1A). Although doxycycline can inhibit mitochondrial translation (*37*), no changes to OXPHOS protein abundance or the ratio of nucleus-encoded to mitochondria-encoded OXPHOS subunits were observed with *Sod2* knockdown (fig. S1B), indicating that this was not a confounding factor between genotypes. SOD2 expression normalized rapidly after birth (when doxycycline was no longer present) as there was no difference in cardiac SOD2 levels between postnatal day 0 (P0) control and iSOD2 mice (fig. S1C).

We next asked whether transient loss of embryonic SOD2 and mitochondrial superoxide induction leads to beneficial adaptations in adult hearts. To test this, we performed bulk RNA sequencing (RNA-seq) of hearts isolated from 6-week-old littermate control (doxycycline, rtTA, and no *Sod2* shRNA) and iSOD2 (doxycycline, rtTA, and *Sod2* shRNA) mice of both sexes. Transient loss of SOD2 during development resulted in persistent transcriptional remodeling, with 201 genes significantly altered in adult iSOD2 hearts (Fig. 1B and table S1). Gene set enrichment analysis (GSEA) revealed an overrepresentation of genes related to mitochondrial metabolism (OXPHOS), ROS detoxification and antioxidant activity (drug metabolism, chemical carcinogenesis, and metabolism of xenobiotics by cytochrome P450), and fat metabolism (fat digestion and absorption and peroxisome proliferator–activated receptor signaling pathway) (Fig. 1C). Up-regulation of OXPHOS genes was similar in males and females, although females showed a more consistent induction of glutathione metabolism transcripts (fig. S1, D and E). Using the TRUSTT2 transcription factor database (*38*), we unbiasedly profiled candidate transcriptional regulators of these responses and identified an enrichment of NRF2 and FOXO3a target genes in iSOD2 mice (fig. S1F). Both NRF2 and FOXO3a are required for mitohormetic life-span extension in invertebrate model systems (*39*, *40*), and we confirmed that they were elevated at the protein level in iSOD2 hearts (Fig. 1D). Accordingly, antioxidant enzyme targets of these transcription factors GSTT1, SOD2, and GSTA1 were also up-regulated (Fig. 1E). Moreover, mitochondrial transcriptional changes translated to an increase in protein abundance of OXPHOS subunits and VDAC, as well as fatty acid β-oxidation enzymes ACADS, HADHA, and ETFDH (Fig. 1F and fig. S1, G to I). Immunofluorescence for HSP60 and cardiac troponin T (cTnT) confirmed this increase in mitochondrial abundance occurred in cardiomyocytes (Fig. 1G). In summary, in response to embryonic matrix superoxide stress, we observed a robust mitohormetic response in adult heart consisting of increased mitochondrial biogenesis and a higher basal antioxidant state.

Given that the heart is quite sensitive to redox and mitochondrial perturbations, we next assessed how embryonic *Sod2* knockdown affects cardiac structure and function in the adult mice. At 6 weeks, iSOD2 mice exhibited both a normal body weight and heart/body weight ratios (Fig. 1H). Histological analysis revealed no aberrant cardiac remodeling at 3 months (Fig. 1I). Reverse transcription quantitative polymerase chain reaction (RT-qPCR) for heart stress transcripts *Nppa* and *Nppb* also indicated the absence of pathological stress responses (Fig. 1J). Last, echocardiographic assessment showed normal ejection fraction and fractional shortening in 3-month-old iSOD2 mice (Fig. 1K). Together, these data demonstrate that transient mitochondrial oxidative stress during embryogenesis via *Sod2* knockdown enhances mitochondrial biogenesis and antioxidant response without inducing major pathological remodeling in the adult heart.

### Mitohormesis preserves mitochondrial content and limits oxidative injury during acute DIC

**Fig. 2.**
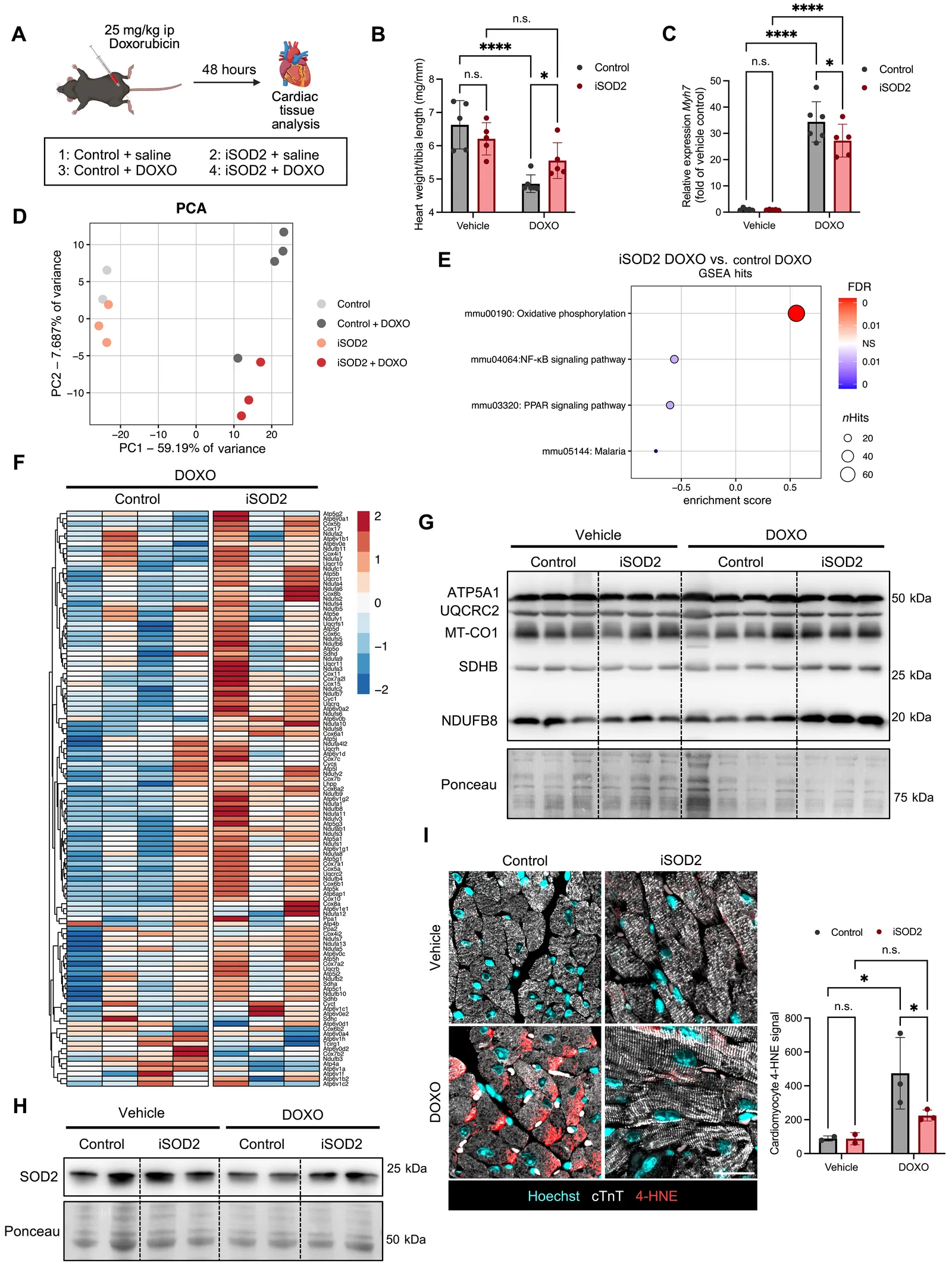
Mitochondrial matrix superoxide–induced mitohormesis preserves mitochondrial content and limits oxidative injury during acute DIC. (**A**) Schematic depicting acute, high-dose DOXO injury regimen. (**B**) Heart weight normalized to tibia length in vehicle- or DOXO-treated control and iSOD2 female mice 48 hours postinjection (*n* = 5 to 6 mice per condition). (**C**) RT-qPCR analysis of cardiac injury marker *Myh7* (normalized to 18*S rRNA*) in cardiac lysates from vehicle- or DOXO-treated control and iSOD2 mice (*n* = 5 to 6 mice per condition). (**D**) Principal components analysis (PCA) plot illustrating the variation in the transcriptome of hearts from vehicle- or DOXO-treated control or iSOD2 mice. (**E**) GSEA for significantly affected transcriptional processes different between control and iSOD2 mice treated with DOXO (*n* = 3 to 4 mice per condition). FDR, false discovery rate; NF-κB, nuclear factor κB. (**F**) Heatmap depicting expression of genes pertaining to mmu00190 OXPHOS pathway comparing control and iSOD2 mice treated with DOXO (*n* = 3 to 4 mice per condition). Color depicts *z*-score. (**G**) Western blot analysis for mitochondrial OXPHOS proteins normalized to total protein confirming the increase in expression in iSOD2 mice treated with DOXO (*n* = 3 to 4 mice per condition). (**H**) Western blot analysis confirming preservation of SOD2 expression in DOXO-treated iSOD2 mice compared with DOXO-treated controls (*n* = 5 to 6 per condition). (**I**) Immunofluorescent staining depicting accumulation of lipid peroxidation [4-hydroxynonenal (4-HNE)] in cardiomyocytes (cTnT^+^ cells) of DOXO-treated controls that is blunted in iSOD2 mice (*n* = 3 mice per condition). Scale bar, 20 μm. Statistical significance is determined using two-way analysis of variance (ANOVA) with Šidák multiple comparisons test correction. *P < 0.05; ****P < 0.0001; n.s., not significant. Histograms represent means ± SEM. Schematic created in BioRender. Donnelly, M. (2026), https://BioRender.com/pt7p85v.

Given the enhanced mitochondrial biogenesis and antioxidant enzyme expression observed in adult iSOD2 hearts, we next asked whether superoxide-mediated mitohormesis is sufficient to protect against acute heart mitochondrial and oxidative injury. The chemotherapeutic DOXO (not to be confused with doxycycline, which was only administered during development to knockdown *Sod2* in utero) induces dose-dependent cardiotoxicity mediated by mitochondrial dysfunction and oxidative stress, which limits its clinical use (*41*). To test whether iSOD2 mice are protected from DOXO-induced cardiac damage, we used an acute, high-dose DOXO administration protocol. Because female iSOD2 mice displayed a more consistent antioxidant response (fig. S1E) and male mice exhibited higher DOXO-induced mortality independent of genotype, subsequent acute experiments were performed in females. Three-month-old control and iSOD2 mice were randomized to a single dose of saline or DOXO (25 mg/kg) injection, and heart tissue was collected after 48 hours (Fig. 2A). Control mice exhibited a significant decrease in heart weight normalized to tibia length following DOXO treatment, indicative of cardiac damage, whereas iSOD2 mice were partially protected from this decrease (Fig. 2B). Expression of the cardiac injury marker *Myh7* was also reduced in iSOD2 hearts compared with controls after acute DOXO administration, supporting an attenuation of cardiac injury (Fig. 2C).

To better understand what drives the mitohormetic protection in this cardiac injury model, we performed bulk RNA-seq of cardiac tissue from control and iSOD2 mice treated with either saline or DOXO (table S2). Principal components analysis revealed that ∼59% of the transcriptional variance was driven by DOXO exposure (Fig. 2D). GSEA confirmed that both genotypes showed canonical DIC signatures, including an underrepresentation of genes involved in regulating cardiac structure and function (dilated cardiomyopathy and hypertrophic cardiomyopathy; fig. S2A). Despite similar pathways being activated in control and iSOD2 mice receiving DOXO, several additional lines of evidence in the gene expression data suggested a partial protection from injury in iSOD2 mice. Acute heart damage in adulthood results in activation of cardiac fetal gene programs (*42*). GSEA confirmed that DOXO-treated iSOD2 mice had significantly decreased expression of genes pertaining to heart development and morphogenesis (fig. S2B). Furthermore, DOXO-treated control and iSOD2 mice clustered distinctly by genotype along PC2 (Fig. 2D). Plotting the top 500 DOXO-responsive genes in control hearts revealed that most changes in gene expression were reduced in iSOD2 mice, consistent with protection from DOXO-induced transcriptional alterations (fig. S2C). Directly comparing DOXO-treated control and iSOD2 groups with GSEA revealed a strong enrichment of OXPHOS genes in iSOD2 mice (Fig. 2, E and F). To further analyze this mitochondrial signature, we interrogated the MitoCarta3.0 database of genes encoding mitochondria-localized proteins (*43*). DOXO injury induced a marked down-regulation of mitochondrial genes that was partially mitigated in iSOD2 mice (fig. S2D). MitoCarta-specific GSEA terms identified OXPHOS complexes (CIV subunits, CV, and OXPHOS assembly factors) and mitochondrial translation processes (mtRNA granules and mitochondrial ribosomes) as the most enriched categories preserved in iSOD2 hearts treated with DOXO (fig. S2E). Western blotting confirmed that iSOD2 mice had increased expression of OXPHOS subunits, particularly NDUFB8, SDHB, and MT-CO1, following DOXO when compared with controls (Fig. 2G and fig. S2F).

Administration of high-dose DOXO (25 mg/kg) in the acute setting has been previously shown to overwhelm antioxidant defenses and significantly decrease *Sod2* transcription (*27*). While control mice had a significant reduction in *Sod2* mRNA and protein expression with DOXO administration, this was completely prevented in iSOD2 mice, consistent with protection from oxidative stress-driven injury (Fig. 2H and fig. S2, G and H). This was accompanied by a significant reduction of DOXO-induced lipid peroxidation measured by 4-hydroxynonenal (4-HNE) staining (Fig. 2I). Together, these results demonstrate that mitohormesis preserves mitochondrial content and antioxidant defenses, thereby tempering acute DOXO-induced oxidative damage and cardiotoxicity.

### Mitohormesis mitigates cardiac dysfunction and remodeling in chronic DIC

**Fig. 3.**
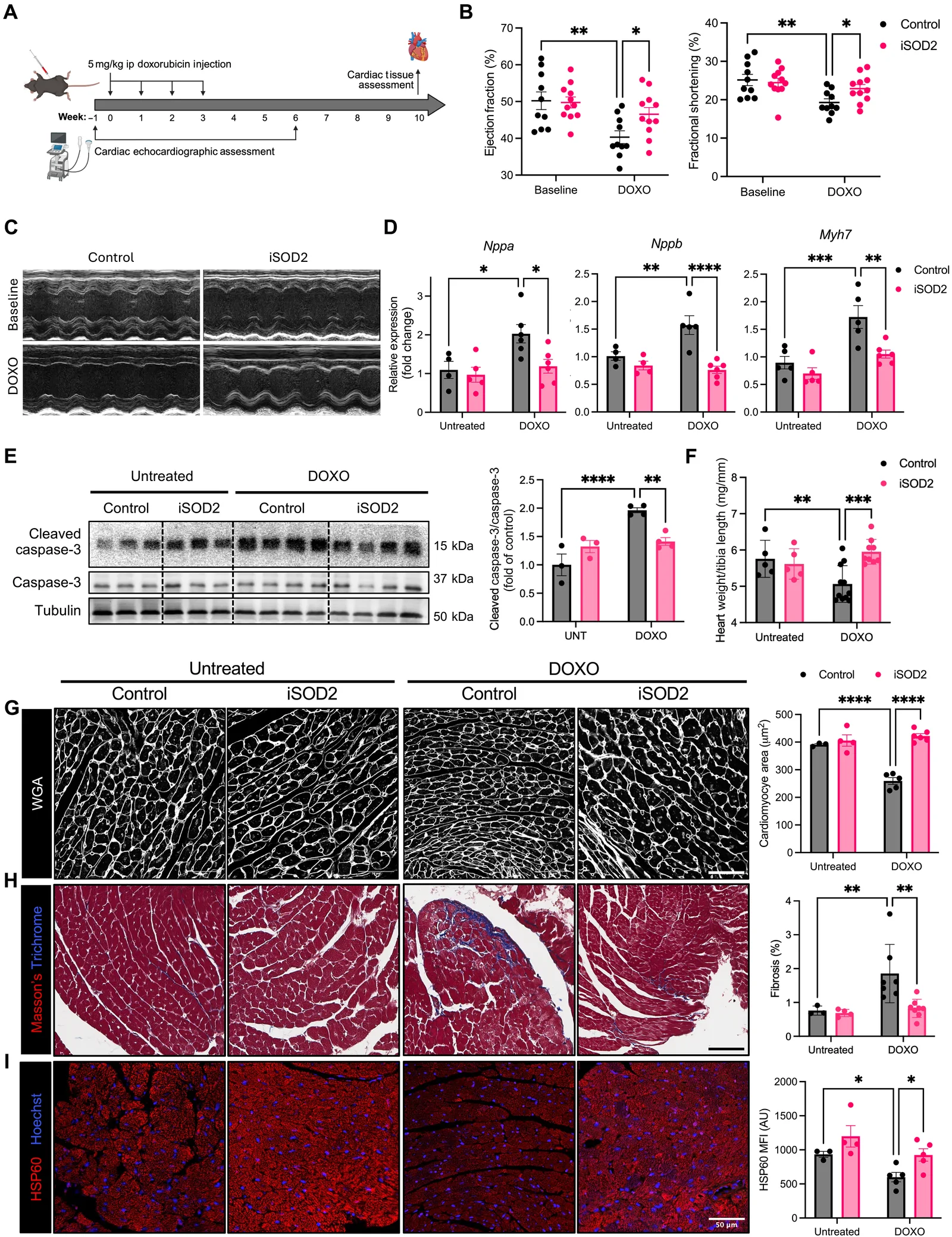
Mitochondrial matrix superoxide–induced mitohormesis mitigates cardiac dysfunction and remodeling in chronic DIC. (**A**) Schematic illustrating dosing regimen and cardiac functional analysis timeline in a model of chronic DIC. (**B**) Ejection fraction and fractional shortening of control (*n* = 9) and iSOD2 (*n* = 8) female mice comparing baseline function to 6 weeks following first DOXO injection. (**C**) Representative left ventricular M-mode echocardiography images of control and iSOD2 mice at baseline and 6 weeks post-DOXO. (**D**) RT-qPCR analysis of heart failure markers *Nppa*, *Nppb*, and *Myh7* (normalized to *Hmbs*) in untreated and DOXO-treated control and iSOD2 mice 10 weeks post–DOXO injection (*n* = 4 to 6 mice per condition). (**E**) Western blotting for apoptosis marker cleaved and total caspase-3 and quantification in untreated and DOXO-treated control and iSOD2 mice (*n* = 3 to 4 mice per condition). (**F**) Cardiac atrophy occurs in control mice treated with DOXO and not iSOD2 mice as depicted by heart weight normalized to tibia length (*n* = 5 to 6 mice per condition). (**G** to **I**) Representative images for (G) wheat germ agglutinin (WGA) staining illustrating cardiomyocyte shrinkage present in control mice receiving DOXO and absent in iSOD2 mice (*n* = 3 to 6 mice per condition), (H) Masson’s trichrome depicting absence of cardiac fibrosis in DOXO-treated iSOD2 mice (*n* = 3 to 8 mice per condition), and (I) preservation of mitochondrial marker HSP60 in iSOD2 mice receiving DOXO (*n* = 3 to 5 mice per condition). Scale bars, 50 μm. Statistical significance is determined using two-way ANOVA with Šidák multiple comparisons test correction. *P < 0.05; **P < 0.01; ***P < 0.001; ****P < 0.0001. Histograms represent means ± SEM. Schematic created in BioRender. Donnelly, M. (2026), https://BioRender.com/c5z50l0.

Encouraged by the differential response observed with acute DOXO injury, we next asked how mitohormesis would affect cardiac function using a more clinically relevant model of chronic DIC. Chronic, low-dose DOXO administration over time is representative of patient dosing regimens and pathophysiology. To model this, baseline cardiac function was measured by echocardiography in 3-month-old female control and iSOD2 mice before initiating a 4-week low dose-DOXO administration protocol. After a 3-week recovery period, cardiac function was reassessed, and hearts were collected at 10 weeks for analysis to allow enough time for remodeling (Fig. 3A). Control mice exhibited the expected DOXO-induced decline in ejection fraction and fractional shortening, whereas iSOD2 mice were partially protected from this functional deterioration (Fig. 3, B and C). This was accompanied by a preservation of body weight, as well as prevention of left ventricular dilation and posterior wall thinning, which were observed in the DOXO-treated controls as expected (fig. S3, A to C). To determine whether cardiac function was preserved in both sexes, we performed echocardiography in male mice at 6 weeks following DOXO treatment. This time point was before the onset of enhanced mortality in male mice. Male iSOD2 mice exhibited a similar preservation of ejection fraction and fractional shortening following chronic DOXO treatment, indicating that this is a conserved protective response in both males and females (fig. S3, D and E).

At 10 weeks following the initial DOXO injection, heart failure–associated gene expression signatures were significantly reduced in iSOD2 mice, consistent with a protection from DIC (Fig. 3D). Similarly, DOXO-induced apoptosis, as assessed by cleaved caspase-3 induction, was markedly increased in control hearts, but mitigated in iSOD2 mice (Fig. 3E). Additionally, the cardiac atrophy characteristic of DIC was present in DOXO-treated controls but alleviated in iSOD2 mice (Fig. 3F). Staining with wheat germ agglutinin (WGA) enabled measurement of cardiomyocyte cell size, which confirmed protection against cardiomyocyte shrinkage typical of DIC (Fig. 3G). Histological analyses revealed a reduction in pathological cardiac remodeling, as iSOD2 hearts exhibited significantly less fibrosis and cardiomyocyte vacuolization (Fig. 3H and fig. S3F). In line with this cardioprotection being driven by mitochondrial and antioxidant effects, iSOD2 mice had preserved mitochondrial content and antioxidant enzyme expression. For example, DOXO-treated iSOD2 mice maintained RNA expression of *Ppargc1b*, *MT-Co1*, and *Sod2* by RT-qPCR (fig. S3G), and immunostaining for HSP60 confirmed that iSOD2 mice were protected from the DOXO-induced decrease in mitochondrial content observed in control mice (Fig. 3I). This was further supported by Western blotting for MT-CO1, which showed the same pattern (fig. S3H). Together, these findings demonstrate that superoxide-mediated mitohormesis protects the heart against a clinically relevant chronic model of DIC by maintaining mitochondrial content, preventing cardiac remodeling, and preserving cardiac function.

### Transient mitochondrial matrix superoxide stress in fibroblasts recapitulates cardiac mitohormetic adaptations

**Fig. 4.**
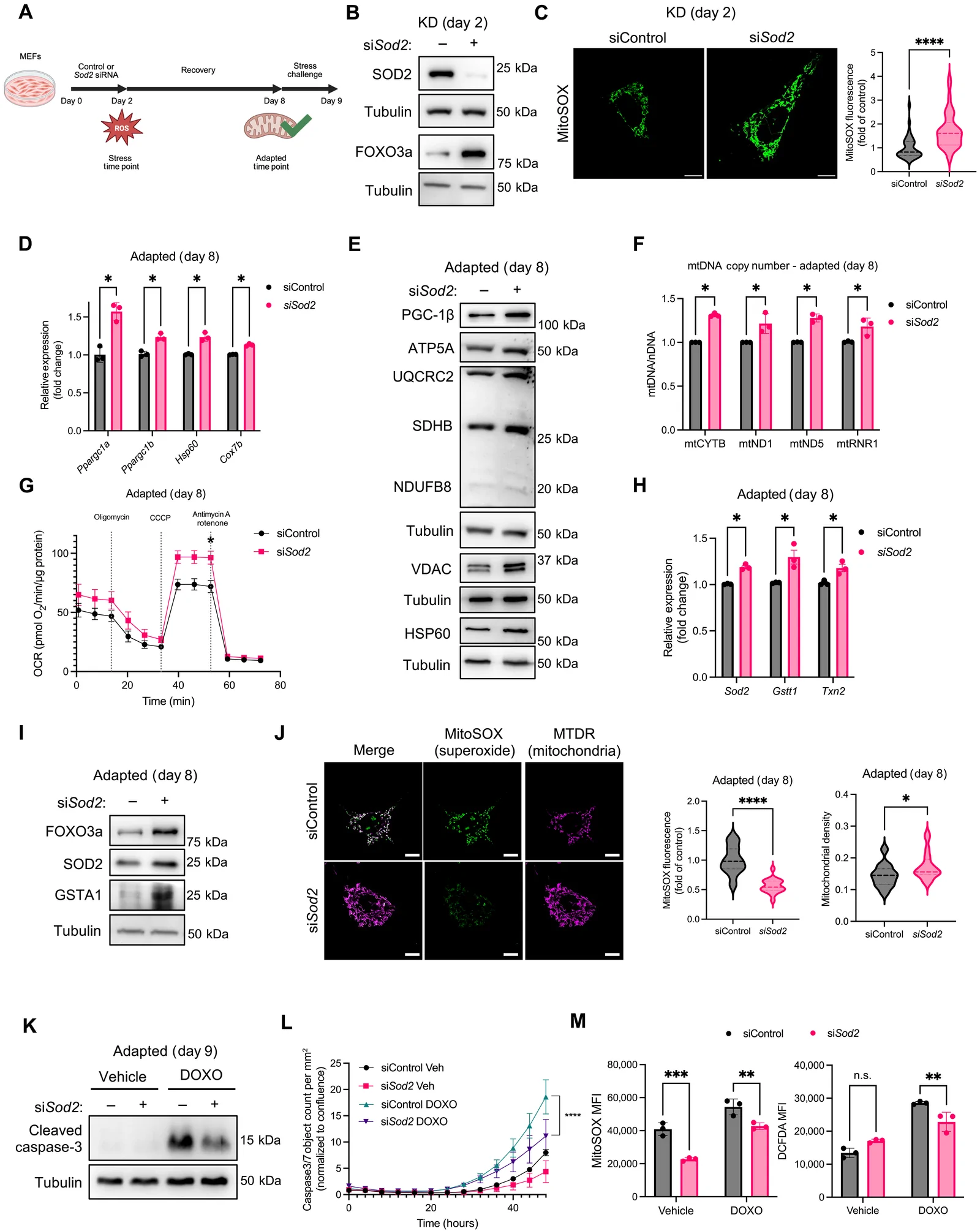
Transient mitochondrial matrix superoxide accumulation in fibroblasts induces mitohormetic adaptations and confers resistance to DOXO-induced cell death. (**A**) Illustration of transient *Sod2* loss, recovery, and stress challenge in mouse embryonic fibroblasts (MEFs). (**B**) Western blot analysis confirming SOD2 loss at 48 hours and induction of mitohormetic stress marker FOXO3a in si*Sod2* MEFs. KD is knockdown. (**C**) Live-cell microscopy measuring mitochondrial superoxide (MitoSOX) at 48 hours of *Sod2* knockdown. (**D**) Gene expression analysis for mitochondrial biogenesis and mitochondrial genes 8 days following *Sod2* loss (adapted) versus negative controls. (**E**) Western blotting confirming increased mitochondrial biogenesis and OXPHOS expression in adapted MEFs. (**F**) Relative mtDNA copy numbers in adapted MEFs normalized to nuclear *B2M*, presented as fold of negative control. (**G**) Mitochondrial oxygen consumption rates (OCRs) in adapted MEFs. Error bars represent SEM of six technical replicates. Graph is representative of three independent biological replicates. (**H**) RT-qPCR analysis of antioxidant enzyme expression in adapted MEFs. (**I**) Western blot analysis confirming increased antioxidant enzyme expression in day 8 adapted MEFs. (**J**) Representative live-cell imaging of mitochondria (MTDR) and superoxide (MitoSOX) in adapted MEFs. (**K**) Representative immunoblot of cleaved caspase-3 in adapted MEFs and negative controls treated with vehicle or DOXO (100 nM) for 24 hours. (**L**) Quantification of IncuCyte imaging of a caspase-3/7 reporter normalized to confluency in adapted MEFs treated with 100 nM DOXO over 48 hours. (**M**) Flow cytometry quantification of mitochondrial superoxide and cellular ROS with MitoSOX and DCFDA, respectively, 24 hours post–100 nM DOXO treatment in adapted MEFs. Significance determined using unpaired *t* test (C to J) and two-way ANOVA with Šidák multiple comparisons test (L and M). *P < 0.05; **P < 0.01; ***P < 0.001; ****P < 0.0001. Histograms represent means ± SEM. Experiments conducted with a minimum *n* = 3 biological replicates unless otherwise stated. Scale bars, 10 μm. Schematic created in BioRender. Donnelly, M. (2026) https://BioRender.com/6dn805j.

Given the robust protection from DOXO in vivo, we sought to generate an in vitro system of mitohormesis to understand the mechanism inducing this phenotype. We showed previously that primary mouse embryonic fibroblasts (MEFs) from iSOD2 mice undergo mitohormesis following doxycycline-induced *Sod2* knockdown in vitro (*23*). To minimize potential confounding effects of doxycycline (not to be confused with DOXO used for the injury model) on mitochondrial translation, we used a strategy of *Sod2* knockdown and recovery with *Sod2*-targeted small interfering RNA (siRNA; Fig. 4A). MEFs transfected with *Sod2* siRNA (si*Sod2*) exhibited decreased *Sod2* mRNA and protein expression (Fig. 4B and fig. S4A). *Sod2* knockdown cells had elevated levels of MitoSOX staining by both microscopy and flow cytometry, consistent with increased superoxide accumulation (Fig. 4C and fig. S4B). DCFDA staining was also lower in *Sod2* knockdown MEFs, consistent with reduced SOD2 conversion of superoxide to hydrogen peroxide (fig. S4B). Western blot analysis revealed an increase in FOXO3a (Fig. 4B), a known mitohormetic transcription factor that we also identified as elevated in iSOD2 hearts (Fig. 1D), confirming an analogous mitohormetic stress in MEFs.

To probe for adaptive mitohormetic effects, we cultured si*Sod2* MEFs for 7 days and examined SOD2 protein reexpression kinetics. By 3 days posttransfection, SOD2 protein levels were reestablished and, at day 7, exhibited a compensatory increase, consistent with an adaptive mitohormetic effect (fig. S4C). Given the mitohormetic mitochondrial biogenesis and antioxidant phenotypes observed in the heart, we tested for these in MEFs having undergone *Sod2* knockdown and recovery at day 8. Transient *Sod2* loss led to a mild, sustained increase in transcription of mitochondrial biogenesis regulators *Ppargc1a* and *Ppargc1b*, as well as *Hsp60* and *Cox7b* as markers of mitochondrial abundance and OXPHOS machinery (Fig. 4D). These results were confirmed by Western blotting, which revealed a significant increase in protein expression of PGC-1β, as well as OXPHOS proteins (ATP5A, UQCRC2, SDHB, and NDUFB8), VDAC, and HSP60 in adapted MEFs (Fig. 4E). In line with enhanced mitochondrial biogenesis, mitochondrial DNA (mtDNA) copy number was significantly increased in MEFs having undergone *Sod2* knockdown (Fig. 4F). Mitochondrial respiration was also elevated in adapted MEFs, with a trend toward increased basal and significantly higher maximal mitochondrial oxygen consumption rates (Fig. 4G and fig. S4D).

We next examined MEFs for the antioxidant signature identified in the hearts of adult iSOD2 mice. Adapted MEFs exhibited a mild increase in transcription of antioxidant enzymes *Sod2*, *Gstt1*, and *Txn2* (Fig. 4H) that correlated with sustained elevation of FOXO3a, as well as SOD2 and GSTA1 at the protein level (Fig. 4I). Immunostaining for FOXO3a confirmed increased nuclear translocation in adapted MEFs, consistent with activation of its function as a transcriptional regulator of antioxidant and stress responses (fig. S4E). Accordingly, despite the higher OXPHOS activity, adapted cells having undergone *Sod2* knockdown had less MitoSOX staining at baseline, consistent with less superoxide production and/or enhanced detoxification (Fig. 4J and fig. S4F). Using MitoTracker Deep Red (MTDR) as a mitochondrial marker further confirmed a mitochondrial biogenesis effect, where cells having experienced *Sod2* knockdown had an increased mitochondrial content (Fig. 4J). Last, like iSOD2 mice having undergone mitohormesis, adapted MEFs were protected from DOXO-induced cell death (again not to be confused with doxycycline) as shown by significantly decreased cleaved caspase-3 (Fig. 4K). This attenuation of apoptosis was confirmed over a 48-hour period with IncuCyte live-cell imaging and a fluorescent caspase-3/7 reporter (Fig. 4L). Adapted SOD2 cells also maintained significantly lower levels of ROS after 24 hours of treatment with DOXO (Fig. 4M). Together, these data support that a transient increase in mitochondrial superoxide via *Sod2* knockdown induces mitohormesis in fibroblasts defined by enhanced mitochondrial biogenesis, antioxidant response, and protection from DOXO-induced cell death, similar to iSOD2 hearts.

### Mitochondrial citrate export mediates matrix superoxide–induced mitohormetic signaling

**Fig. 5.**
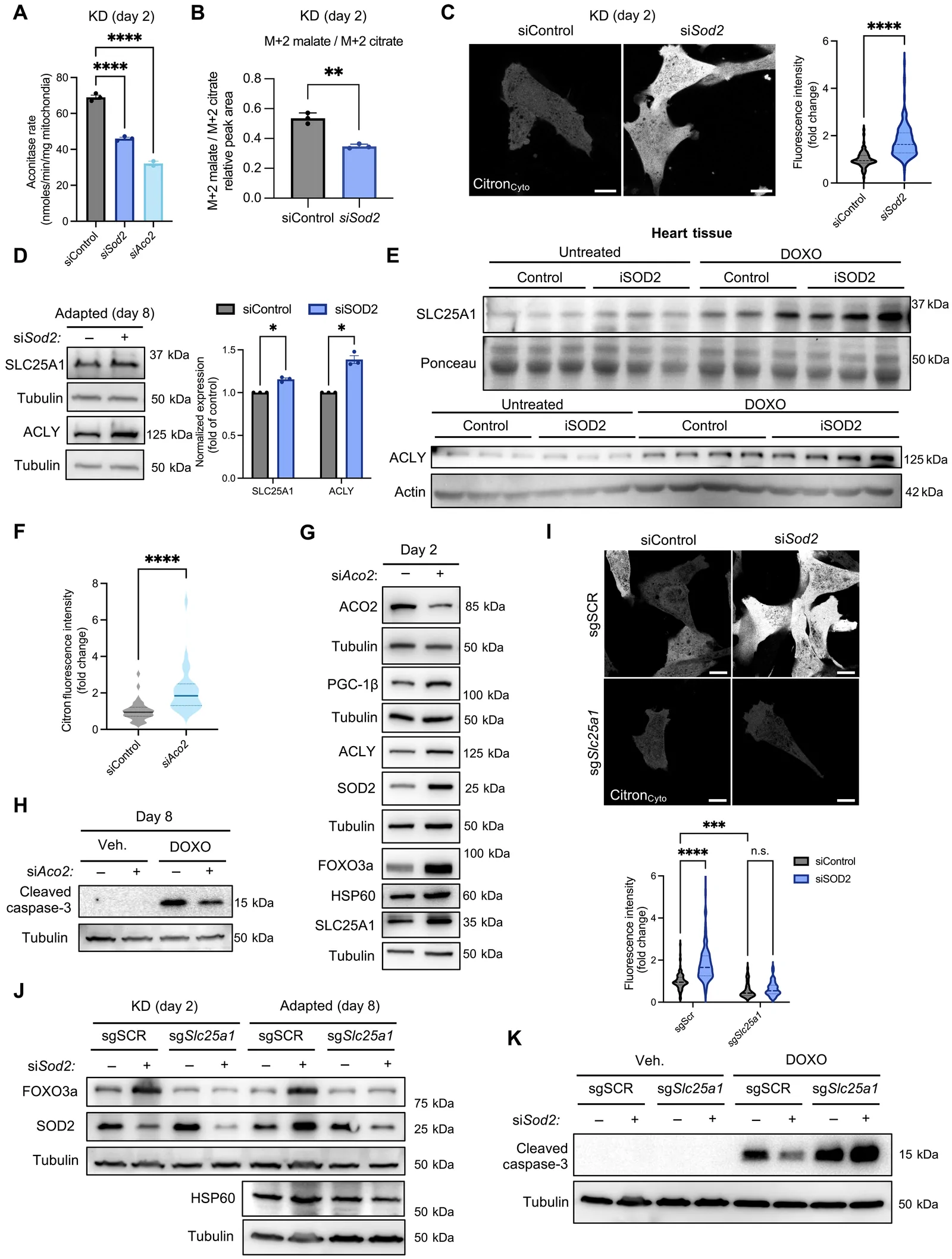
Mitochondrial citrate export mediates mitochondrial matrix superoxide–induced mitohormetic signaling. (**A**) Mitochondrial aconitase (ACO2) rate (nmoles per minute per microgram of mitochondria) in isolated mitochondrial from MEFs 48 hours post–*Sod2* or *Aco2* knockdown. KD is knockdown. Graph is technical replicates of one biological replicate (*n* = 2). (**B**) Total molar abundance of glucose-derived malate M+2 relative to citrate M+2 after 48 hours of *Sod2* knockdown. (**C**) Representative live-cell microscopy and fluorescence quantification of a cytosolic targeted citrate biosensor, Citron_Cyto_, 48 hours post–*Sod2* knockdown (*n* = 91 control, *n* = 180 si*Sod2* cells, and *n* = 3 biological replicates). (**D**) Representative Western blots and quantification for citrate metabolic proteins in adapted MEFs, 8 days post–*Sod2* knockdown. (**E**) Western blot analysis for citrate metabolic proteins in heart lysates of either untreated or 10-week post–chronic DOXO–treated control and iSOD2 mice (*n* = 3 to 4 mice per condition). (**F**) Quantification of increased Citron_Cyto_ fluorescence 48 hours post–*Aco2* knockdown (*n* = 55 cells per condition and *n* = 3 biological replicates). (**G**) Western blot analysis of increased mitohormetic signature 48 hours post–*Aco2* knockdown. (**H**) Western blot of adapted day 8 si*Aco2* MEFs displaying protection from apoptosis following 100 nM DOXO for 24 hours. (**I**) Representative live-cell imaging and quantification of sgSCR or sg*Slc25a1* MEFs expressing Citron_Cyto_ following *Sod2* knockdown (*n* = 50 to 250 cells per condition and *n* = 3 biological replicates). (**J**) Western blot analysis of antioxidant and mitochondrial proteins in sgSCR or sg*Slc25a1* MEFs 2 and 8 days post–*Sod2* knockdown. (**K**) Western blot analysis depicting loss of protection from 100 nM DOXO in sg*Slc25a1* MEFs 8 days post–*Sod2* knockdown. Significance determined using one-way ANOVA with Dunnett’s multiple comparisons test (A), unpaired *t* test (B to F), and two-way ANOVA with Šidák multiple comparisons test (I to K). *P < 0.05; **P < 0.01; ***P < 0.001; ****P < 0.0001; n.s., not significant. Histograms represent means ± SEM and all experiments were conducted with a minimum *n* = 3 biological replicates unless otherwise stated. Scale bars are all 10 μm.

As superoxide is membrane impermeable, we sought to identify the retrograde signal responsible for mitohormesis induced by increased mitochondrial matrix superoxide. Superoxide inhibits the activity of iron-sulfur cluster containing enzymes, including the TCA cycle protein aconitase (ACO2) (*44*). In fact, ACO2 activity is commonly used to assay mitochondrial ROS-mediated damage and oxidative stress. We therefore isolated mitochondria and confirmed that 48 hours of *Sod2* knockdown decreased ACO2 activity (similar in degree to *Aco2* knockdown), without affecting total ACO2 protein abundance (Fig. 5A and fig. S5A). Metabolites can act as retrograde signals from mitochondria (*3*), and, because ACO2 converts citrate to isocitrate in the TCA cycle, a reduction in its activity would be expected to increase mitochondrial citrate levels. However, mitochondrial citrate can also be transported to the cytosol via the mitochondrial citrate transporter (SLC25A1) and subsequently metabolized by ATP citrate lyase (ACLY). We therefore hypothesized that mitochondrial superoxide stress signals through ROS-mediated inhibition of ACO2 and altered citrate flux. Metabolic tracing with [U-^13^C] glucose can provide insight into the fate of citrate by examining the proportion of labeled citrate (M+2) to labeled (M+2) malate (*45*). When processed by ACLY in the cytosol, M+2 citrate will generate an unlabeled oxaloacetate, which will subsequently be metabolized to unlabeled malate and thus dilute the pool of M+2 malate generated from TCA cycle (fig. S5B). Accordingly, cytosolic citrate processing by ACLY in lieu of metabolism by ACO2 will decrease the ratio of M+2 malate/M+2 citrate. To test this, we incubated control and si*Sod2* knockdown MEFs for 1 hour with [U-^13^C] glucose. There was no change in the total abundances of glycolytic intermediates, TCA cycle metabolites, or amino acids between conditions after 48 hours of knockdown (fig. S5C). Control and si*Sod2* cells also exhibited no difference in the fraction of M+2-labeled citrate, indicating no change in glucose entry into TCA cycle (fig. S5D). However, consistent with decreased ACO2 activity, there was significantly reduced labeling of TCA cycle intermediates downstream of ACO2, including fumarate and malate (fig. S5D). The mole percent enrichment of total malate labeling was also significantly decreased in si*Sod2* cells and, in line with cytosolic processing of citrate by ACLY, the ratio of M+2 malate/M+2 citrate was significantly reduced with *Sod2* loss (Fig. 5B and fig. S5E). This efflux of mitochondrial citrate was confirmed using a cytosol-targeted citrate biosensor (Citron_Cyto_) (*46*), revealing a marked increase in cytosolic citrate levels in *Sod2-*deficient cells (Fig. 5C). Consistent with cytosolic citrate metabolism, both SLC25A1 and ACLY were increased at the protein level in adapted MEFs having previously experienced *Sod2* knockdown (Fig. 5D). SLC25A1 and ACLY were also up-regulated in heart tissue following chronic DOXO treatment, with a preferential enhancement in iSOD2 mice compared with DOXO-treated controls (Fig. 5, E and F). These findings, together with recent reports implicating ACLY in cardioprotection (*47*, *48*), suggest that citrate export and metabolism by ACLY may contribute to the mitohormetic stress protection.

To determine whether mitochondrial citrate export is sufficient to induce mitohormesis, we directly silenced *Aco2*. As expected, *Aco2* knockdown decreased aconitase activity in isolated mitochondria and increased cytosolic citrate to a degree comparable to SOD2 loss (Fig. 5, A and F, and fig. S5G). Consistent with citrate redistribution and processing in the cytosol, both SLC25A1 and ACLY were up-regulated in si*Aco2* cells (Fig. 5G). These cells also exhibited a molecular signature of mitohormesis characterized by increased PGC-1β, HSP60, FOXO3a, and SOD2 expression (Fig. 5G and fig. S5H). Cells having undergone transient *Aco2* knockdown retained a mitohormetic memory, displaying reduced DOXO-induced apoptosis 8 days following transfection, as shown by decreased cleaved caspase-3 (Fig. 5H). To confirm that efflux of mitochondrial citrate is required for the mitohormetic adaptation observed with either *Sod2* or *Aco2* loss, we generated MEFs lacking the mitochondrial citrate transporter, SLC25A1, using CRISPR-Cas9 (fig. S5I). Genetic disruption of SLC25A1 completely blocked cytosolic citrate accumulation following *Sod2* knockdown (Fig. 5I) and abolished all adaptive responses (e.g., FOXO3a, SOD2, and HSP60 induction were lost and cells failed to resist DOXO-induced apoptosis) (Fig. 5, J and K). Similarly, the increase in SOD2 and FOXO3a induced by *Aco2* loss was prevented in SLC25A1 knockout MEFs, as well as the protection from DOXO (fig. S5, J and K). Last, consistent with cytosolic citrate accumulation inducing a mitohormetic protection, supplementing exogenous citrate for 24 hours resulted in a dose-dependent increase in the amount of cytosolic citrate and protection from DOXO-induced cleaved caspase-3 expression (fig. S5, L and M). Together, these findings demonstrate that metabolic signaling by mitochondrial citrate efflux is both necessary and sufficient for mitohormesis, thereby inducing adaptive mitochondrial and antioxidant responses and conferring protection from DOXO.

### Mitohormesis increases histone acetylation via mitochondrial citrate export

**Fig. 6.**
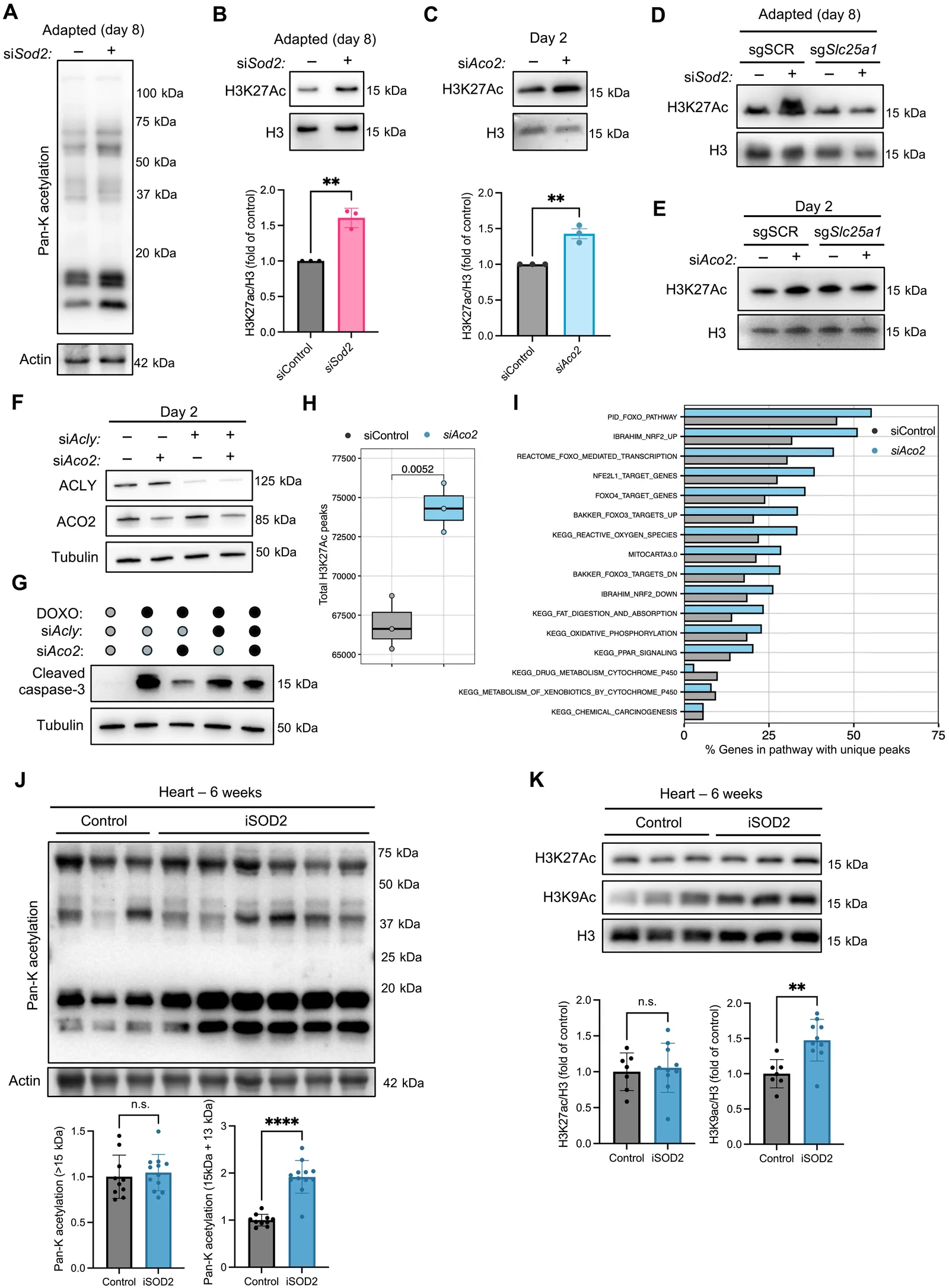
Mitochondrial matrix superoxide–induced mitohormesis increases histone acetylation via mitochondrial citrate export. (**A**) Representative Western blot illustrating increased lysine acetylation at proteins of 15 and 11 kDa in adapted MEFs 8 days posttransfection with *Sod2* siRNA versus negative siRNA controls. (**B** and **C**) Representative Western blot for increased H3K27Ac normalized to total H3 in (B) adapted MEFs 8 days following transfection with *Sod2* targeting siRNA and (C) adapted MEFs 2 days following *Aco2* knockdown compared with negative controls. (**D** and **E**) Representative Western blots illustrating SLC25A1 dependence of both (D) *Sod2* and (E) *Aco2*-mediated mitohormetic increase in histone acetylation. (**F**) Representative Western blot illustrating co-knockdown of ACO2 and ACLY compared with negative controls. (**G**) Representative Western blot depicting that the ACO2-mediated protection from DOXO (100 nM for 24 hours) is partially ACLY dependent. (**H**) Quantification of the number of total H3K27Ac peaks identified from CUT&RUN analysis of cells 48 hours following transfection with control or *Aco2* targeting siRNA. (**I**) Quantification of the percent of genes in relevant mitohormetic gene sets of interest gaining unique H3K27Ac peaks from CUT&RUN data following 48 hours of *Aco2* knockdown. (**J**) Western blot analysis of pan-lysine acetylation in heart lysates from 6-week-old littermate control or iSOD2 mice illustrating the increase in histone acetylation induced by mitohormetic embryonic *Sod2* silencing (*n* = 10 control and *n* = 12 iSOD2 mice). (**K**) Representative Western blot illustrating the increase in H3K9Ac, but not H3K27Ac, in 6-week-old iSOD2 hearts compared with littermate controls (*n* = 7 control and *n* = 10 iSOD2 mice). All experiments conducted in three biological replicates unless otherwise stated. Statistical significance determined using unpaired *t* test. **P < 0.001; ****P < 0.0001; n.s., not significant. Histograms represent means ± SEM.

We next sought to understand how mitochondrial citrate export mediates mitohormesis and “memory” to a transient mitochondrial oxidative stress. In invertebrate model systems, mitohormesis extends life span via coordinated epigenetic remodeling, including a loss of repressive histone methylation and gain of histone acetylation (*49*, *50*). Because cytosolic citrate metabolized by ACLY also generates acetyl-CoA, a substrate for histone acetylation, we hypothesized that increased histone acetylation driven by citrate efflux is a feature of superoxide-mediated mitohormesis. Western blot analysis in day 8 adapted MEFs having previously experienced *Sod2* knockdown revealed a modest global increase of pan-lysine acetylation (Fig. 6A). However, there was a pronounced elevation of lysine acetylated proteins of 15 and 11 kDa, the molecular weights of H3 and H4, respectively (Fig. 6A). Accordingly, bulk H3K27Ac, a marker of active enhancers, was also increased in adapted si*Sod2* MEFs (Fig. 6B). In support of citrate efflux driving this change, direct knockdown of *Aco2*, as well as supplementation with exogenous citrate, was sufficient to increase global H3K27Ac levels (Fig. 6C and fig. S6A). Conversely, blocking mitochondrial citrate export by knocking out SLC25A1 completely prevented the increase in H3K27Ac seen with si*Sod2* and si*Aco2*-induced mitohormesis (Fig. 6, D and E, and fig. S6B). To confirm that acetyl-CoA generation from citrate by ACLY is required for mitohormetic protection, we performed co-knockdown of *Aco2* and *Acly* for 48 hours, followed by DOXO treatment. Accordingly, loss of ACLY significantly blunted the mitohormetic protection seen with *Aco2* knockdown (Fig. 6, F and G). Last, supplying cells with acetate, a second source of acetyl-CoA via ACSS2 metabolism, was sufficient to increase H3K27Ac (fig. S6, C and D). Acetate supplementation induced the mitohormetic signature including FOXO3a, SOD2, and VDAC and protected MEFs from DOXO, highlighting a role for acetyl-CoA downstream of citrate and acetate in mitohormesis (fig. S6, E and F).

Acetylation of H3K27 will facilitate gene transcription by relaxing condensed chromatin (*50*). To understand how mitochondria-derived citrate influences histone acetylation patterns at specific genomic loci, we performed CUT&RUN for H3K27Ac 48 hours following *Aco2* knockdown. Globally, loss of ACO2 resulted in a significant increase in the number of H3K27Ac peaks compared with controls, consistent with the increase in H3K27Ac observed by Western blotting (Fig. 6H). Given the effects on mitochondrial biogenesis and antioxidant response in our mitohormetic model, we quantified H3K27Ac peak accumulation unique to each genotype in all genes annotated to these pathways. Consistently, loss of ACO2 resulted in an accumulation of unique H3K27Ac peaks when compared with controls in these gene sets, including FOXO3 and NRF2 targets and genes annotated in MitoCarta3.0 (*43*) and ROS processing (Fig. 6I). Across these gene sets of interest, cells with *Aco2* knockdown had a significant increase in the percent of genes in each pathway with unique H3K27Ac peaks (fig. S6G). Together, these genomic data support that mitochondria-derived citrate increases global H3K27Ac deposition, including at many genes relevant to the mitohormetic response.

Last, we confirmed that increased histone acetylation was also a conserved feature of superoxide-mediated mitohormesis in vivo. Liver tissue from 6-week-old iSOD2 mice exhibited significantly elevated H3K27Ac compared with littermate controls (fig. S6G). In heart tissue, there was no change in global lysine acetylation patterns, but a similar increase in bands at 15 and 11 kDa as seen in MEFs with *Sod2* knockdown (Fig. 6H). However, bulk H3K27Ac was unchanged in iSOD2 hearts (Fig. 6I). Instead, H3K9Ac was elevated, indicating that there are tissue-specific responses to chromatin remodeling induced by mitohormesis (Fig. 6I). Notably, H3K9Ac accumulation at the promoters of antioxidant genes has previously been linked to cardioprotection (*51*). These data establish that mitohormetic signaling induces cell-type–specific histone acetylation patterns in vitro and in vivo, which is dependent on mitochondrial citrate.

## DISCUSSION

Pioneering work in *S. cerevisiae*, *C. elegans*, and *D. melanogaster* illustrates that inhibition of mitochondrial OXPHOS and antioxidant responses can extend lifespan, often through increases in mitochondrial ROS that act as mitohormetic signals (*8*, *9*, *21*, *52*, *53*). Here, we demonstrate that mitohormesis driven by mitochondrial matrix superoxide accumulation elicits a mitohormetic program in the adult mouse heart, which protects against mitochondrial and oxidative injury in acute and chronic models of DIC. This superoxide stress is communicated via mitochondrial citrate efflux to regulate histone acetylation and mitochondrial and antioxidant enzyme expression.

While some mitohormetic adaptations appear broadly conserved, others exhibit cell type and tissue specificity (*54*). We build upon this using our iSOD2 model, providing evidence that adapted hearts activate mitochondrial biogenesis and antioxidant transcriptional programs similar to those observed in liver (*23*), but with distinct upstream transcriptional regulators and target-gene patterns. For example, NRF2 was enriched in both tissues, but its canonical target *Nqo1* was selectively induced in liver. Conversely, increased expression of FOXO3a and its transcriptional target SOD2 was unique to heart. These observations suggest that noncanonical activation of these transcription factors and subsequent mitohormetic signaling is tailored to specific tissue environments, perhaps based on distinct metabolic and redox constraints. This could be particularly important for heart tissue, which requires a redox-optimized ROS balance to maintain mechano-energetic coupling for proper cardiac function (*35*, *55*). In line with this, inducing mitochondrial biogenesis and antioxidant pathways simultaneously with mitohormesis did not result in overt cardiac injury. Chromatin remodeling offers an additional layer of transcriptional regulation and has been implicated in mitohormetic life-span extension in invertebrate model organisms, albeit with distinct histone residues being modified depending on the species (*49*, *50*, *56*). Accordingly, we observe distinct histone acetylation patterns in adapted liver and heart (H3K27Ac versus H3K9Ac), which may help shape differential transcriptional responses to the same initial mitohormetic stress.

DOXO remains a highly effective chemotherapeutic agent for both solid tumors and hematological malignancies, yet its clinical utility is limited by dose-dependent cardiotoxicity driven by mitochondrial damage and oxidative injury, resulting in heart failure (*29*, *41*, *57*). Once diagnosed with DIC, the 5-year survival is less than 50%, highlighting the critical need for therapeutic options that prevent its onset (*58*). Here, we provide robust in vivo evidence that the simultaneous activation of endogenous mitochondrial biogenesis and antioxidant pathways by mitohormetic signaling confers protection in acute and clinically relevant chronic models of DIC. Our findings provide additional evidence that mitochondrial dysfunction and oxidative stress contribute to DIC pathophysiology. Previous attempts to enhance mitochondrial biogenesis or antioxidant defenses with genetically modified mouse models resulted in cardiac dysfunction (*32*–*34*). In contrast, our data suggest that coordinated activation of both responses simultaneously using endogenous mitohormetic signaling cascades does not affect cardiac function under basal physiological conditions and may be therapeutic in DIC. This aligns with previous studies showing varying degrees of cardioprotection due to ROS-mediated mitohormetic-like signaling (*59*–*65*). For example, the mitohormetic effect that we observe shares features with ischemic preconditioning, where transient ischemia triggers intrinsic protective mechanisms that prevent future injury (*66*). Notably, our findings show that a brief, targeted induction of mitochondrial superoxide is sufficient to induce protection, which may offer more focused interventions in lieu of global tissue hypoxia. More broadly, these results provide proof of principle that mitochondrial stress signaling enhances disease resistance in mammals and thus provides a framework for potential health span–promoting interventions.

Although inducing mitochondrial ROS during embryogenesis is not a feasible therapeutic approach, delineating pathways downstream of mitohormesis may reveal actionable targets in adulthood. Using a fibroblast model of mitohormesis, we identify a redox-sensitive mitochondrial retrograde signaling mechanism whereby superoxide-mediated inhibition of aconitase (ACO2) redirects mitochondrial citrate to the cytosol through SLC25A1. Cytosolic citrate then acts as a superoxide-sensitive second messenger, fueling ACLY-derived acetyl-CoA production and subsequent histone acetylation, which together promote the observed mitohormetic mitochondrial and antioxidant responses. Multiple lines of evidence support the relevance of this pathway in vivo. In *C. elegans*, *aco-2* loss extended life span in a screen for longevity genes and protected from infection via antioxidant gene transcription (*67*, *68*). Moreover, dietary citrate supplementation prolongs life span in *D. melanogaster*, suggesting this signaling axis may be evolutionarily conserved (*69*). SLC25A1 also regulates mitochondrial gene expression during heart development by modulating H3K9Ac near gene promoters, while CBP/p300-dependent histone acetylation is required for mitohormetic life-span extension in *C. elegans* (*50*, *70*). Two recent studies highlight a cardioprotective role for ACLY, where cardiomyocyte specific loss depresses cardiac function at baseline and in response to pressure overload (*47*, *48*). Last, this may be conserved in humans, as patients with hypertrophic cardiomyopathy and heart failure exhibit decreased ACLY expression. Consistent with this, we find that SLC25A1 and ACLY are preferentially up-regulated in mitohormetically adapted hearts subjected to chronic DOXO, raising the possibility that enhanced citrate-driven acetylation contributes to DIC protection. Notably, a recent study demonstrated that chronic, supraphysiologic citrate elevation via whole-body ACO2 deficiency in adulthood caused extensive kidney damage (*71*). These data highlight the importance of considering dosing and timing, which can determine whether citrate acts as a metabolic stressor or beneficial signaling molecule. Similar to the concept of mitohormesis, whereby transient, mild stress is required for beneficial adaptation, we illustrate that a temporary alteration in citrate flux, rather than accumulation of the metabolite, activates mitochondrial and antioxidant programs. Therefore, future studies will be required to determine whether targeted citrate-driven signaling can be therapeutically engaged in the adult heart. Last, while our data highlight a role for H3K27Ac deposition at mitochondrial and antioxidant genes, non-histone acetylation likely also participates in mitohormetic signaling. For example, FOXO3 and PGC-1α are themselves regulated by acetylation, and cytosolic acetyl-CoA concentrations directly regulate mitophagy (*72*, *73*). Defining the full spectrum of acetylation targets remodeled by citrate-derived acetyl-CoA will be critical in understanding the contribution of this signaling arm to mitohormesis across tissues and distinct mitochondrial stressors.

## MATERIALS AND METHODS

### Animal studies

C57BL/6J mice were purchased from the Jackson Laboratory (strain 000664). The iSOD2 mice were generated as previously described (*23*). Briefly, iSOD2 mice harbor an rtTA2 under the *Rosa26* promoter and a shRNA targeting the *Sod2* gene downstream of a tetracycline response element (TRE) expressed at the *Col1a1* locus.

Embryonic SOD2 knockdown was achieved by breeding wild-type C57BL/6J female mice with male iSOD2 mice homozygous for the rtTA2 and heterozygous for the TRE-SOD2-shRNA. This allowed for generation of intrauterine littermate controls heterozygous for the rtTA2 but lacking the TRE-SOD2-shRNA and experimental iSOD2 mice harboring one copy of the TRE-SOD2-shRNA. Mice were monitored for vaginal plugs daily and when identified dams were supplied with doxycycline-containing rodent chow (625 mg/kg; Harlan Teklad TD.01306) to achieve intrauterine SOD2 knockdown. The doxycycline chow was refreshed weekly until pups were born, at which time the doxycycline chow was replaced with normal chow and experimental pups were either collected between 12 and 24 hours after birth (P0) or aged to 6 weeks for liver and heart harvest. Embryonic heart tissue was also collected at E17.5 to confirm SOD2 knockdown. Food and water intake were available ad libitum. All animal procedures were conducted in accordance with the Institutional Animal Care and Use Committee at the Salk Institute for Biological Studies under animal use protocol 17-00044.

### DOXO administration

Female and male iSOD2 and littermate control mice at 10 to 12 weeks of age were used for DOXO experiments. DOXO was prepared by diluting in LAL water to a stock solution (10 mg/ml) and used within 14 days. On the day of injection, the stock solution was diluted in phosphate-buffered saline (PBS) to an appropriate working concentration. For the acute DOXO injury model, female mice were randomly assigned to a one-time vehicle (PBS) or DOXO (25 mg/kg body weight) intraperitoneal (ip) injection and euthanized at 48 hours. In the chronic DOXO-injury model, female and male mice were given DOXO injections (5 mg/kg ip) weekly for 4 consecutive weeks (total dose, 20 mg/kg). Mice were aged for 10 weeks following initial DOXO injection and subsequently euthanized. For all experiments, heart tissue was dissected and cut in transverse sections, where the same region of cardiac tissue was used for downstream analyses across mice.

### Cell culture

Immortalized MEFs were purchased from American Type Culture Collection. MEFs were cultured in Dulbecco’s modified Eagle’s medium (DMEM) supplemented with 10% (v/v) fetal bovine serum (FBS) and maintained at 37°C with 5% CO_2_. Cells were kept in culture for a maximum of 20 passages and tested for mycoplasma monthly (MycoAlert Mycoplasma Detection Kit).

### Histology and immunofluorescence staining

Freshly dissected heart ventricular tissue was cut into 1-mm transverse sections, fixed in 4% paraformaldehyde (PFA) overnight at 4°C, and dehydrated in alcohol before paraffin embedding and sectioning at 6-μm thickness. Tissue sections were dewaxed and rehydrated through xylene and ethanol washes and subsequently stained with hematoxylin and eosin (H&E) and Masson’s trichrome or processed for immunofluorescence. Antigen retrieval was performed using 10 mM citrate buffer (pH 6), and sections were blocked with 10% goat serum in 1% bovine serum albumin (BSA) in PBS for 1 hour, followed by primary antibody treatment overnight at 4°C in a humidified chamber. Secondary antibody incubation was performed with fluorescence-conjugated Alexa Fluor antibodies (1:500) in accordance with the primary antibody species and counterstained with Hoechst (1:1000) for 1 hour. Cardiomyocyte cross-sectional area was measured using WGA Alexa Fluor 647 conjugate (1:100) that was added at the time of secondary antibody incubation. Sections were mounted on slides with ProLong Glass Antifade and subsequently imaged.

For cell immunofluorescence, MEFs were seeded on glass coverslips coated with fibronectin. The following day, cells were fixed at 37°C in a prewarmed 4% PFA solution for 15 min. Following three washes with PBS, cells were permeabilized in 0.1% (v/v) Triton X-100 in PBS for 10 min at room temperature and blocked in 0.22-μm-filtered 1% (w/v) BSA for 1 hour. Primary antibodies diluted in 1% BSA were incubated overnight at 4°C according to the manufacturer’s dilutions. The next day, cells were washed three times with PBS and incubated with fluorescence-conjugated Alexa Fluor secondary antibodies (1:500) diluted in blocking buffer for 1 hour at room temperature. Coverslips were washed three times in PBS and counterstained with Hoechst (Thermo Fisher Scientific) at 1:1000 for 10 min, followed by three PBS washes. Coverslips were mounted using ProLong Glass Antifade mounting medium and subsequently imaged.

Histology images were acquired with Leica Aperio AT2 Scanscope, and fixed immunofluorescent images were collected using a Plan-Apochromat 63×/1.4 numerical aperture (NA) oil objective on an inverted Zeiss LSM 880 confocal microscope with Airyscan. Images were collected as *z*-stacks, and representative images are shown as maximum intensity *z*-projections. Airyscan data were processed using ZEN Black (Zeiss version 2.3) and subsequently visualized and analyzed using Fiji software [National Institutes of Health (NIH), version 2.9.0]. Fibrosis area was analyzed from the Masson’s trichrome sections using the Fiji plugin Color Deconvolution 2. Cardiomyocyte vacuolization was quantified from H&E sections as % of cardiomyocytes with >1 vacuole. Scoring of six independent fields of view from each heart was used in quantification of cardiomyocyte vacuolization, cardiomyocyte cross-sectional area, HSP60 intensity, and 4-HNE intensity.

### RNA-seq analysis

Total RNA was extracted from heart tissue using the RNeasy Plus Universal Mini Kit and quantified using the Qubit RNA Broad Range Assay Kit. Before library preparation, the RNA quality was assessed by RNA ScreenTape assay. Libraries were prepared using the Illumina TruSeq Stranded Total RNA Gold and sequenced using the NovaSeq X Plus platform. For all experiments, raw reads were quality checked with FastQC v0.11.8. Experiments containing adaptor sequences were trimmed with Trim Galore v0.4.4_dev. Reads were aligned to the mm10 reference genome with star aligner v2.5.3a and converted to gene counts with HOMER’s analyzeRepeats.pl script (*74*, *75*). Gene counts were normalized and queried for differential expression using DESeq2 v1.30.0 (*76*). For each pairwise comparison, genes with fewer than 10 total raw counts across all samples were discarded before normalization, and genes with an absolute log_2_ fold chance of >0.25 and a false discovery rate–corrected *P* value of ≤0.05 were pulled as significant. Pathways were queried for treatment-specific functional enrichment using GSEA (in WebGestaltR v0.4.4) using rank-ordered log_2_ fold change values (*77*). Raw sequencing data are available at the Gene Expression Omnibus (GEO) under accession codes: GSE313130 for 6-week control versus iSOD2 hearts and GSE313129 for acute DOXO-treated hearts, respectively.

### Western blotting

For heart tissue, the same transverse portion of the left ventricle was homogenized directly in 1× Laemmli supplemented with 1× protease/phosphatase inhibitor cocktail using QIAGEN metal PowerBead tubes and a Biomedicals FastPrep-24 lysis system. For MEFs, cells were lysed directly in 1× Laemmli buffer supplemented with 1× protease/phosphatase inhibitor cocktail. Protein was quantified using a Pierce detergent compatible Bradford assay with BSA standards. Proteins (15 to 40 μg) were separated by SDS–polyacrylamide gel electrophoresis, followed by transfer to a polyvinylidene difluoride membrane (MilliporeSigma). Membranes were subsequently stained with Ponceau S (Thermo Fisher Scientific) to assess loading before blocking in 5% BSA in tris-buffered saline supplemented with 0.1% Tween 20 (TBST) at room temperature for 1 hour. Primary antibodies were applied to the membrane overnight at 4°C, followed by washing with TBST and incubation with horseradish peroxidase–conjugated secondary antibody in 5% BSA in TBST for 1 hour at room temperature. Membranes were developed with ProSignal Femto ECL Reagent (Genesee Scientific) on the ChemiDoc MP Imaging System (Bio-Rad Libraries).

### Echocardiography

Control and iSOD2 mice of both sexes were depilated with Nair body cream on the chest 1 day before echocardiography assessment. Anesthesia induction was conducted by placing mice in a chamber with inhaled isoflurane at 3%. During echocardiographic assessment, light anesthesia was supplied using a nose cone with inhaled isoflurane at 1%. Mice were continuously monitored on a heated stage during echocardiography. Light anesthesia was administered to maintain a heart rate greater than 400 beats per min during experimental measurements. Transthoracic echocardiography was conducted using the VisualSonics Vevo 3100 system and a MX550D imaging transducer. The user was blinded to mouse treatment group and genotype. Left ventricular papillary muscle was used as an anatomical landmark in B mode, and parasternal short axis images were acquired over five cardiac cycles in M mode. Left ventricular posterior wall thickness end-systole (LVPW_s_) and left ventricular internal diameter end-systole (LVID_s_) were obtained from M-mode tracings. Left ventricular ejection fraction (LVEF%) was calculated as the percent of blood ejected from the left ventricle during systole relative to the total end-diastolic volume. Fractional shortening (FS%) was obtained by measuring the relative change in left ventricular diameter during systole relative to diastole (LVID_d_ − LVID_s_)/LVID_d_. Echocardiography was analyzed using the Vevo LAB analysis software.

### Reverse transcription quantitative polymerase chain reaction

Total RNA was extracted from the same transverse portion of left ventricle tissue across mice. The tissue was homogenized in lysis buffer supplied from the RNeasy Plus Universal Mini Kit using metal QIAGEN PowerBead tubes and a Biomedicals FastPrep-24 lysis system. RNA was isolated using the RNAeasy Plus Universal Mini Kit according to the manufacturer’s instructions. Total RNA was extracted from MEFs using the RNeasy Plus Mini Kit per manufacturer’s protocol. Reverse transcription of 2 μg RNA was used to generate cDNA with the High-Capacity cDNA Kit. Equal amounts of cDNA and the indicated primers were used for RT-qPCR using FAST SYBR Green Master Mix on the CFX384 Touch Real-Time PCR Detection System. For each biological replicate, three technical triplicates were assessed and the relative mRNA expression per gene was calculated with the 2^−ΔΔCt^ method. All samples were normalized to Hydroxymethylbilane synthase (*Hmbs*) except for RNA isolated from mice undergoing acute DOXO injury, which was normalized to 18*S* as *Hmbs* expression changed with DOXO treatment. For each sample, the fold change in experimental mRNA expression was plotted relative to its respective controls, which were given a value of 1. Primer sequences are found in table S3.

### siRNA and DNA transfection

For siRNA transfection, 1 × 10^6^ cells were seeded in a 100-mm dish and allowed to adhere overnight. The next day, transfection of either *Sod2* targeting or *Aco2* targeting siRNAs was conducted using Lipofectamine RNAiMax according to the manufacturer’s instructions to achieve a final concentration of 60 or 50 nM, respectively. Cells were collected and used for downstream experiments at two time points: 48 hours following transfection to assess knockdown and stress phenotypes and 8 days following transfection to probe adaptive effects to transient knockdown. Sequences are found in table S3. To assess relative cytosolic citrate concentration, MEFs were transfected with 2 μg of cytomegalovirus (CMV)–Citron1 using Lipofectamine 3000 transfection reagent at a ratio of 2:1 [Lipofectamine 3000 (microliters):nucleic acid (micrograms)] according to the manufacturer’s protocol. Fresh medium was supplied 6 hours posttransfection, and cells were subsequently knocked down with either *Sod2*, *Aco2*, or *Acly* targeting siRNA. Measurements were conducted 48 hours after transfection.

### Determination of mitochondrial ROS by flow cytometry

MEFs from their respective experimental paradigms were counted to ensure that an equal number (300,000 to 400,000) per condition were used for all experiments. Cells were equilibrated in serum-free DMEM at 37°C for 30 min before staining. To measure both mitochondrial and whole-cell ROS, MEFs were stained in both 5 μM MitoSOX and 10 μM H2-DCFDA, respectively, in the dark for 30 min at 37°C. MitoSOX and H2-DCFDA staining was confirmed in each experiment by treating cells with 50 μM menadione for 30 min as a positive control. Following incubation with dyes, cells were washed in fluorescence-activated cell sorting (FACS) buffer (PBS with 2% v/v FBS). Cells were subsequently resuspended in FACS buffer supplemented with Zombie Violet Viability dye (1:1000; BioLegend) to detect and remove dead cells in downstream analysis. All imaging was conducted using a FACSymphony A3 Cell Analyzer (BD Biosciences). Following gating on live, singlet cells, at least 10,000 events were collected for downstream analysis using FlowJo v10. Mean fluorescence intensity is plotted for each dye.

### Determination of mitochondrial content, ROS and cytosolic citrate by live-cell imaging

All live-cell imaging experiments were conducted in eight-well chamber slides. MEFs from all conditions were plated at 12,000 cells per well and allowed to adhere overnight. The next day, cells were incubated for 30 min with 5 μM MitoSOX to assess mitochondrial ROS and 100 nM MTDR to assess mitochondrial content. As a positive control for MitoSOX, cells from every experiment were incubated with 50 μM menadione for 30 min and monitored for an increase in MitoSOX fluorescence. Cells were washed three times in PBS and switched to live-cell imaging media with phenol red–free DMEM and supplemented with 10% (v/v) FBS. For cytosolic citrate quantification, cells were transfected with CMV-Citron1, followed by the appropriate siRNA condition. After 24 hours, cells were seeded at a density of 12,000 cells per well and allowed to adhere overnight before live-cell imaging the following day. To measure cytosolic citrate with exogenous citrate supplementation, cells were transfected with CMV-Citron1 and 24 hours later treated with exogenous sodium citrate (500 μM to 5 mM), followed by a 24-hour incubation before live-cell imaging. All live-cell imaging experiments were conducted using a Plan-Apochromat 63×/1.4 NA oil objective lens on an inverted Zeiss 880 LSM confocal microscope with Airyscan. Cells were imaged at 37°C, 5% CO_2_, and controlled humidity using the environmental control system. Representative live-cell images are shown as single planes.

### mtDNA copy number

MEF cell pellets from day 8 control or day 8 si*Sod2* conditions were resuspended in 50 mM NaOH and incubated at 95°C for 30 min. NaOH was neutralized by adding 1/10 volume of 1 M tris-HCl (pH 8.0). Isolated DNA was diluted to 10 ng/μl in deoxyribonuclease-free DepC-treated water and underwent qPCR analysis using primers against mtDNA: *mt-CYTB*, *mt-ND1*, *mtND5*, and *mtRNR1* and nuclear DNA: *B2m*. Three technical replicates were analyzed per biological replicate, and each mtDNA-encoded sequence was normalized against the nuclear control (*B2m*). Relative mtDNA copy number (mtDNA/nDNA) was analyzed using a 2^−ΔΔCt^ method and plotted as the fold change relative to the control mtDNA copy number, which was given a value of 1. Sequences are found in table S3.

### Seahorse extracellular flux analysis

For control and day 8 si*Sod2* adapted MEFs, oxygen consumption rates (OCRs) were measured using a Seahorse XFe96 Extracellular Flux Analyzer (Agilent). MEFs were seeded at a density of 15,000 cells per well in Agilent Seahorse XFe96 microplates with eight technical replicates per biological replicate. Before beginning the assay, cells were equilibrated in serum-free Seahorse XF base medium lacking phenol red (Agilent) and supplemented with 10 mM glucose, 2 mM glutamine, and 1 mM sodium pyruvate for 1 hour at 37°, in a normoxic incubator. Mitochondrial respiration was determined by the consecutive addition of 1.5 μM oligomycin A, 3.5 μM CCCP, and 0.5 μM rotenone and antimycin A. OCR was normalized to protein isolated from each respective well and measured using the Pierce BCA Protein Assay Kit. Data were normalized and analyzed with the Agilent Seahorse Wave Controller 2.6.1 software.

### Cell treatments

To test whether mitohormetically adapted cells were protected from injury, day 8 negative control and day 8 si*Sod2* or si*Aco2* adapted MEFs were cultured in 100 nM DOXO for 24 hours and collected for downstream analysis. For experiments using the IncuCyte Cell Proliferation Assay and caspase-3/7 reporter, MEFs were cultured in 100 nM DOXO for 48 hours. To test the effects of metabolites on mitohormetic phenotypes, MEFs were treated with increasing concentrations (500 μM to 5 mM) of sodium citrate or (1 to 10 mM) sodium acetate for 24 to 48 hours and replenished daily. Following 24 hours of treatment with the indicated metabolite, cells were refreshed with each metabolite and cotreated with 100 nM DOXO for 24 hours.

### IncuCyte cell proliferation and caspase-3/7 reporter assays

Adapted si*Sod2* MEFs at day 8 and their respective negative controls were seeded at a density of 5000 cells per well in a 96 well plate and allowed to adhere overnight. The next day, cells were treated with 100 nM DOXO and CellEvent caspase-3/7 green reagent at a 1:1000 concentration to assess active caspase-3/7 activity. Plates were placed in the Sartorius IncuCyte S3 Live Cell Analysis System at ×10 magnification for 48 hours, and phase-contrast images were acquired every 4 hours. Green caspase-3/7–positive events were reported relative to cell confluency calculated using the IncuCyte S3 software. All conditions were performed in three technical replicates with three independent biological replicates.

### Mitochondria isolation and aconitase activity

To specifically assess the activity of mitochondrial aconitase, enriched mitochondrial fractions were prepared by hypotonic shock and differential centrifugation. MEFs were transfected for 48 hours with either negative control, *Sod2*, or *Aco2* siRNA. Cells were collected from four, fully confluent 100-mm dishes per condition and washed once in ice-cold PBS. Cells (1:20) were taken for protein isolation to assess knockdown efficiency, while the remaining cells were resuspended in an 8:1 buffer:pullet (v:v) ratio of hypotonic buffer [83 mM sucrose and 10 mM Mops (pH 7.2)]. Cells were incubated for 2 min in hypotonic buffer on ice to permit cell swelling before performing homogenization with 30 strokes in a glass-Teflon homogenizer. Immediately following homogenization, the same volume of hypertonic buffer [250 mM sucrose and 30 mM Mops (pH7.2)] was added. Two centrifugations at 1000*g* for 5 min at 4°C was conducted to remove nuclei and unbroken cells. Mitochondria were pelleted by centrifugation at 9500*g* for 10 min at 4°C and washed once in buffer A [320 mM sucrose, 1 mM EDTA, and 10 mM tris-HCl (pH7.4)]. Protein quantification was determined with the BCA Protein Assay Kit with BSA standards.

Enriched mitochondrial fractions were then immediately used to assess aconitase activity using the Aconitase Activity Assay Kit (Colorimetric). Mitochondria were freeze thawed twice on dry ice to lyse mitochondria, and samples were prepared according to the manufacturer’s instructions. Briefly, isolated mitochondria from each condition were resuspended to 5 mg/ml in ice-cold aconitase preservation solution. Samples were further diluted to 18 μg of mitochondria/50 μl in assay buffer. Activity buffer was prepared using 1/25 volume isocitrate and 1/100 volume manganese in assay buffer. Sample (50 μl) was then incubated with 200 μl of activity buffer, and the conversion of isocitrate to cis-aconitate by aconitase was monitored every 30 s over 30 min as an increase in absorbance at 240 nM. Aconitase rate was determined as the average change in absorbance over time and corrected for background and concentration of mitochondria loaded. Conditions were conducted in technical duplicate or triplicate, and the experiment was repeated with two separate biological replicates.

### ^13^C-labeled glucose trace metabolomics

For metabolic tracing experiments, MEFs were transfected with either negative control or *Sod2* targeting siRNA. The day before tracing, cells were seeded at 250,000 cells in a six-well plate and allowed to adhere overnight. The next day, at 48 hours following transfection, MEFs were washed with PBS and cultured in tracing media comprising glucose-free DMEM supplemented with 10% (v/v) FBS and the labeled [U-^13^C] glucose to achieve a final concentration of 25 mM for 1 hour before collection. Following labeling with [U-^13^C] glucose, cells were washed in an ice-cold 0.9% NaCl solution, and metabolites were extracted in an ice-cold 60:40 methanol/water mixture followed by transfer to Eppendorf tubes. Norvaline (0.5 μg) was added as a standard to each sample, at which point samples were vortexed for 5 min and 10% was collected for protein quantification using BCA assay. To the remaining sample, chloroform was added in a 1:1 ratio (v/v) to separate polar and organic phases. Samples were vortexed for 5 min and underwent centrifugation at 21,000*g* for 5 min at 4°C. The upper aqueous phase containing polar metabolites was collected and transferred to gas chromatography–mass spectrometry (GC-MS) vials and dried overnight in a CentriVap at 4°C.

Dried polar metabolites were processed for GC-MS. Polar metabolites were derivatized using a Gerstel Multipurpose Sample (MPS 2XL). Samples were first derivatized via addition of 15 μl of 2% (w/v) methoxyamine hydrochloride (MP Biomedicals) in pyridine and incubated for 60 min at 45°C. Next, samples were silylated with addition of 15 μl of *N-tert*-butyldimethylsily-*N*-methyltrifluroacetamide with 1% *tert*-butyldimethylchlorosilane (Regis Technologies) followed by incubation at 45°C for 30 min. The derivatized samples were analyzed using gas GC-MS on a DB-35MS column (30 m by 0.25 mm internal diameter by 0.25 μm, Agilent) installed in an Agilent 7890B GC system integrated with an Agilent 5977a MS. Samples were subsequently injected at an initial oven temperature of 100°C and held for 1 min, then ramped at 3.5°C/min up to 255°C, followed by 15°C/min up to 320°C, and held for 3 min. Electron impact ionization was used and the instrument scanned over an mass/charge ratio range between 100 and 650. Quantification of metabolites and isotopologue distribution analysis were performed with in-house MATLAB scripts that include correction for natural isotope abundance (*78*). Peaks for compounds of interest were isolated and normalized to both the internal standard (norvaline) and protein abundance from BCA assay for three replicates. Ratios of M+2 malate/M+2 citrate represent the ratio of relative isotopically corrected peak areas for each labeled metabolite.

### CUT&RUN

CUT&RUN was performed as previously described (*79*). For H3K27Ac, 0.5 × 10^6^ cells were counted, washed in PBS, and collected in a V-bottom 96-well plate via centrifugation. The cells were washed twice in antibody buffer [20 mM Hepes (pH 7.5), 150 mM NaCl, 0.5 mM spermidine, 0.01% digitonin, 1× complete protease inhibitor EDTA-free, and 2 mM EDTA] and incubated with the H3K27Ac antibody (1:100) diluted in antibody buffer for 1 hour on ice. Cells were washed twice in buffer 2 [20 mM Hepes (pH 7.5), 150 mM NaCl, 0.5 mM spermidine, 0.01% digitonin, and 1× complete protease inhibitor EDTA-free] and subsequently incubated with EpiCypher pA/G-MNase (SKU 15-1116) at 1:20 dilution in buffer 2 for 1 hour at 4°C. Cells were then washed in buffer 2 twice and resuspended in calcium buffer (buffer 2 with 1 mM CaCl_2_) to activate the MNase fusion protein. Cells were incubated for 30 min on wet ice and then 2× stop solution [20 mM EDTA, 4 mM EGTA, and ribonuclease A (100 μg/ml) in buffer 2] was added, and cells were incubated for 14 min at 37°C to release cleaved chromatin. The supernatants were collected by centrifugation, and DNA was collected with a QIAGEN MinElute kit.

Libraries for CUT&RUN were prepared with a NEBNext Ultra II kit (New England Biolabs) according to the manufacturer’s protocol. The barcoded libraries were quantified on an Agilent TapeStation. Samples were mixed at an equal molar ratios and pooled libraries were sequenced using the Element Aviti platform for 50–base pair pair-ended reads.

CUT&RUN samples were processed with the nf-core/cutandrun pipeline v3.2.2 with settings: --genome mm10 -profile singularity --skip_fastqc true --skip_igv true --normalisation_mode CPM --peakcaller macs2. Condition-specific peaks were identified as any consensus peak found in (*n −* 1) samples with no overlapping peak in the alternative condition. Nearest-gene annotations were identified with Homer’s annotatePeaks.pl; intergenic peaks greater than 5 kb away from a TSS were excluded from target gene tabulation. Raw sequencing data are available at the GEO under accession code GSE337993.

### RNP assembly and nucleofection

Multiguide single guide RNA (sgRNA)/Cas9 ribonucleoprotein (RNP) complexes were assembled using 6 μl of target sgRNA mix (30 μM) with 1 μl of SpCas9 2NLS nuclease (20 μM) and 18 μl of Nucleofector Solution and Supplement (4.5:1 ratio of solution to supplement). This mixture was incubated at room temperature for 10 min to 1 hour during cell preparation. MEFs (2 × 10^5^) were washed with PBS and resuspended in Nucleofector Solution plus Supplement and then combined with the precomplexed RNP. The RNP-cell mixture was moved to a Nucleocuvette strip and electroporated using a Lonza 4D-Nucleofector. Immediately following electroporation, cells were resuspended in prewarmed DMEM and transferred to a six-well plate. Following a 3-day incubation, protein was isolated from electroporated cells and immunoblot analysis of protein extracts were conducted to determine editing efficiency.

### Statistical analysis

All statistical analysis was conducted with GraphPad Prism 9 software (v10.4.2). For each experiment, the sample size and description of bar plots are present in the corresponding figure legend. All experiments were conducted at least three times, unless otherwise indicated. Statistical significance between two groups were determined using unpaired, two-tailed Student’s *t* test. Statistical differences between three or more groups were determined by analysis of variance [one-way or two-way analysis of variance (ANOVA)] with either Dunnett’s multiple comparisons test or Šidák multiple comparisons test correction, respectively. Data are represented as mean values, and error bars are SEM unless otherwise indicated. *P* values of less than 0.05 were considered significant and displayed in the figures.
