# Supplementary material for: Mitochondrial superoxide–induced mitohormesis is mediated by citrate and cardioprotective
Source: Sci Adv. 2026 Sep 4;12(36):eaef8132. doi: 10.1126/sciadv.aef8132 (PMC13544202; doi:10.1126/sciadv.aef8132)
Supplement: Supplementary file 1 [file sciadv.aef8132_sm.pdf]

Science Advances
AAAS

Supplementary Materials for

Mitochondrial superoxide–induced mitohormesis is mediated by citrate and cardioprotective

Matthew P. Donnelly *et al.*

Corresponding author: Gerald S. Shadel, gshadel@salk.edu

Sci. Adv. **12**, eaef8132 (2026)
DOI: 10.1126/sciadv.aef8132

The PDF file includes:

Figs. S1 to S6
Legends for tables S1 to S3

Other Supplementary Material for this manuscript includes the following:

Tables S1 to S3

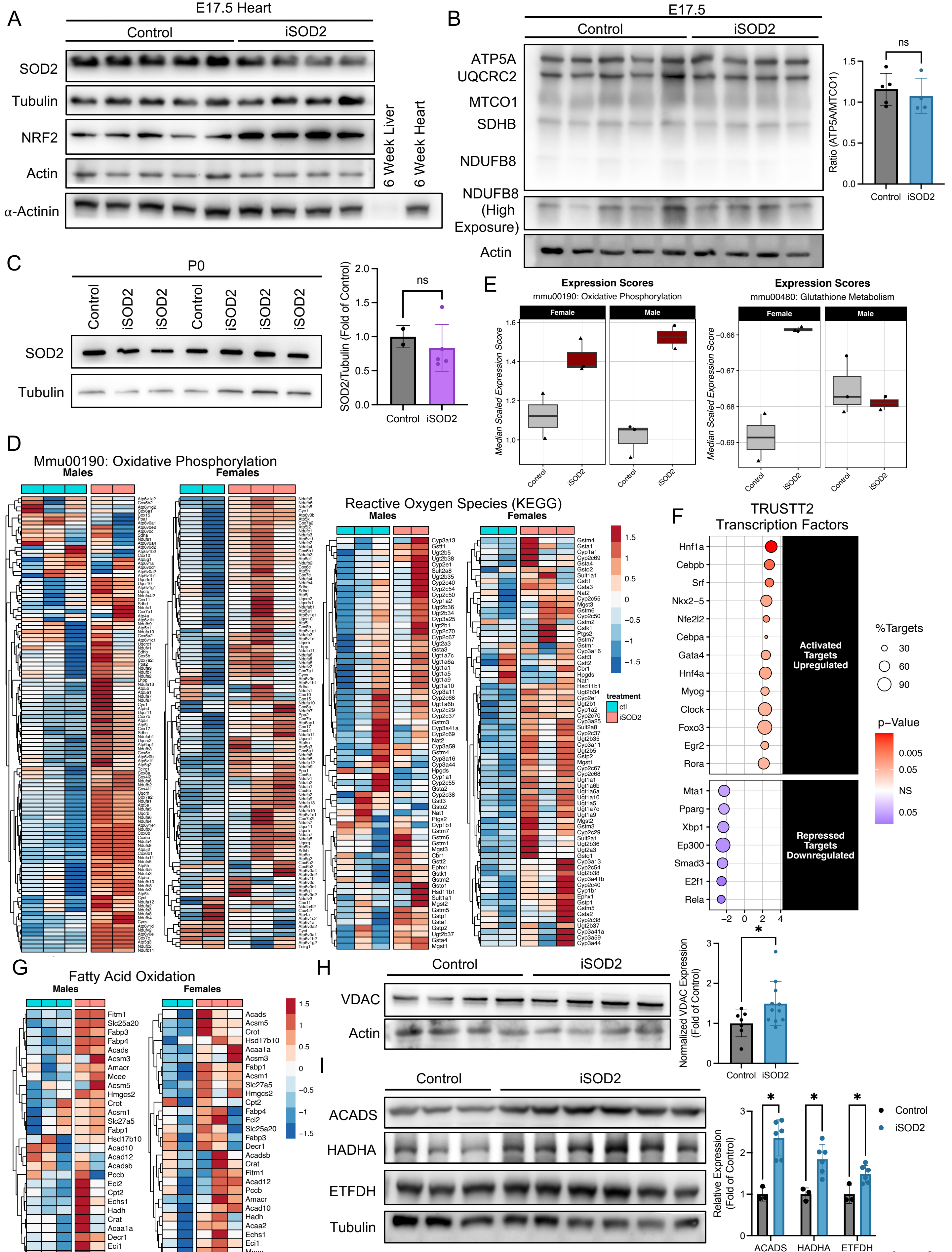

A
E17.5 Heart
Control
iSOD2
SOD2
Tubulin
NRF2
Actin
α-Actinin
6 Week Liver
6 Week Heart
B
E17.5
Control
iSOD2
ATP5A
UQCRC2
MTCO1
SDHB
NDUFB8
NDUFB8 (High Exposure)
Actin
Ratio (ATP5A/MTCO1)
ns
C
P0
SOD2
Tubulin
SOD2/Tubulin (Fold of Control)
D
Mmu00190: Oxidative Phosphorylation
Males
Females
Reactive Oxygen Species (KEGG)
treatment
ctl
iSOD2
E
Expression Scores
mmu00190: Oxidative Phosphorylation
mmu00480: Glutathione Metabolism
Female
Male
Median Scaled Expression Score
F
TRUSTT2
Transcription Factors
Activated Targets Upregulated
Repressed Targets Downregulated
%Targets
p−Value
NS
G
Fatty Acid Oxidation
H
VDAC
Normalized VDAC Expression (Fold of Control)
I
ACADS
HADHA
ETFDH
Relative Expression (Fold of Control)

fig. S1

Fig. S1. Effects of embryonic matrix superoxide accumulation in the heart and adaptive responses in adulthood

(A) Immunoblot analysis and densitometry of SOD2 loss and NRF2 induction as a readout of oxidative stress in E17.5 littermate control and iSOD2 hearts. Cardiac tissue isolation is confirmed by α-actinin expression that is absent in adult liver tissue (n=4-5 mice per condition). **(B)** Immunoblot analysis confirming no change in OXPHOS protein abundance in E17.5 hearts from iSOD2. The ratio of a nuclear-encoded (ATP5A) to mitochondrial-encoded (MTCO1) subunit is quantified to illustrate no confounding effect on mitochondrial translation by doxycycline in embryonic heart (n=4-5 mice per condition). **(C)** Immunoblot analysis illustrating return of SOD2 protein expression in hearts from P0 iSOD2 mice within 24 h of doxycycline removal (n=2-5 mice per condition). **(D)** Heatmaps illustrating changes in OXPHOS and ROS enzyme gene expression from bulk RNA-seq of littermate 6-week control and iSOD2 hearts. Color represents Z-score (n=5 mice per condition). **(E)** Gene expression scores pertaining to OXPHOS and glutathione metabolism genes from RNA-seq split by sex and genotype. **(F)** TRUSTT2 transcription factor analysis illustrating enrichment of NRF2 (*Nfe2l2*) and FOXO3 target genes in iSOD2 hearts compared with littermate controls (n=5 mice per condition). **(G)** Heatmap illustrating changes in fatty acid oxidation gene expression from bulk RNA-seq of littermate 6-week control and iSOD2 hearts. Color represents Z-score (n=5 mice per condition). **(H)** Representative immunoblot and quantification depicting the increase in VDAC expression in iSOD2 vs. control mice (n=7-11 mice per condition). **(I)** Immunoblot analysis and densitometry of proteins involved in mitochondrial fatty acid oxidation in 6-week-old control and iSOD2 mice (n=3-6 mice per condition). Statistical significance determined by unpaired Student's *t*-test. *$p<0.05$, ***$p<0.001$, ns is not significant. Histograms represent mean ± SEM.

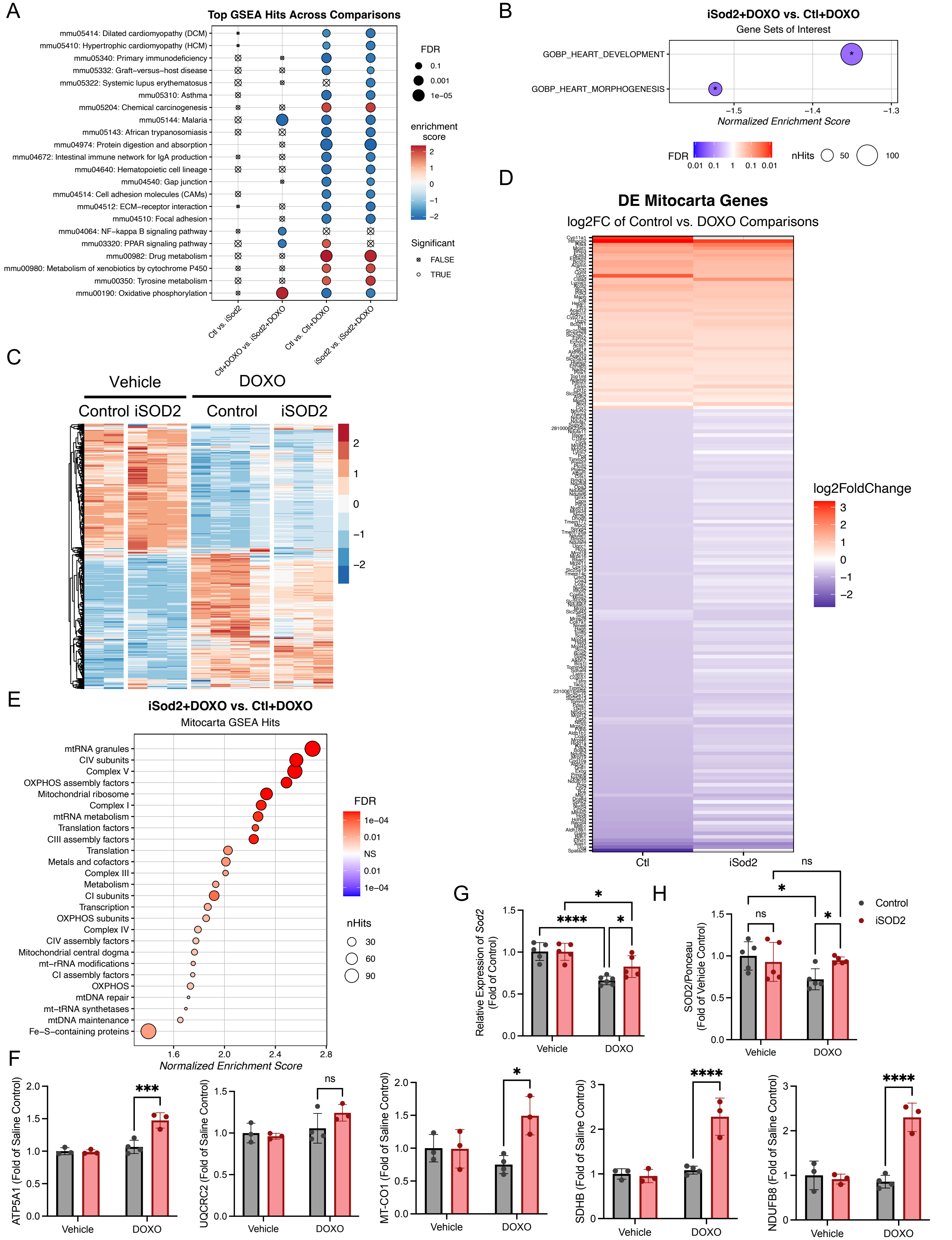
A
Top GSEA Hits Across Comparisons
mmu05414: Dilated cardiomyopathy (DCM)
mmu05410: Hypertrophic cardiomyopathy (HCM)
mmu05340: Primary immunodeficiency
mmu05332: Graft−versus−host disease
mmu05322: Systemic lupus erythematosus
mmu05310: Asthma
mmu05204: Chemical carcinogenesis
mmu05144: Malaria
mmu05143: African trypanosomiasis
mmu04974: Protein digestion and absorption
mmu04672: Intestinal immune network for IgA production
mmu04640: Hematopoietic cell lineage
mmu04540: Gap junction
mmu04514: Cell adhesion molecules (CAMs)
mmu04512: ECM−receptor interaction
mmu04510: Focal adhesion
mmu04064: NF−kappa B signaling pathway
mmu03320: PPAR signaling pathway
mmu00982: Drug metabolism
mmu00980: Metabolism of xenobiotics by cytochrome P450
mmu00350: Tyrosine metabolism
mmu00190: Oxidative phosphorylation
Ctl vs. iSod2
Ctl+DOXO vs. iSod2+DOXO
Ctl vs. Ctl+DOXO
iSod2 vs. iSod2+DOXO
FDR
0.1
0.001
1e−05
enrichment score
2
1
0
−1
−2
Significant
FALSE
TRUE
B
iSod2+DOXO vs. Ctl+DOXO
Gene Sets of Interest
GOBP_HEART_DEVELOPMENT
GOBP_HEART_MORPHOGENESIS
−1.5
−1.4
−1.3
Normalized Enrichment Score
FDR
0.01 0.1 1 0.1 0.01
nHits
50
100
C
Vehicle
DOXO
Control iSOD2
Control
iSOD2
2
1
0
−1
−2
D
DE Mitocarta Genes
log2FC of Control vs. DOXO Comparisons
log2FoldChange
3
2
1
0
−1
−2
Ctl
iSod2
E
iSod2+DOXO vs. Ctl+DOXO
Mitocarta GSEA Hits
mtRNA granules
CIV subunits
Complex V
OXPHOS assembly factors
Mitochondrial ribosome
Complex I
mtRNA metabolism
Translation factors
CIII assembly factors
Translation
Metals and cofactors
Complex III
Metabolism
CI subunits
Transcription
OXPHOS subunits
Complex IV
CIV assembly factors
Mitochondrial central dogma
mt−rRNA modifications
CI assembly factors
OXPHOS
mtDNA repair
mt−tRNA synthetases
mtDNA maintenance
Fe−S−containing proteins
1.6
2.0
2.4
2.8
Normalized Enrichment Score
FDR
1e−04
0.01
NS
0.01
1e−04
nHits
30
60
90
G
Relative Expression of Sod2 (Fold of Control)
****
*
*
Vehicle
DOXO
H
SOD2/Ponceau (Fold of Vehicle Control)
ns
*
ns
*
Vehicle
DOXO
Control
iSOD2
F
ATP5A1 (Fold of Saline Control)
***
UQCRC2 (Fold of Saline Control)
ns
MT-CO1 (Fold of Saline Control)
*
SDHB (Fold of Saline Control)
****
NDUFB8 (Fold of Saline Control)
****
Vehicle
DOXO

fig. S2

Fig. S2. Matrix superoxide-induced mitohormesis protects against acute doxorubicin-mediated transcriptional alterations, mitochondrial damage, and oxidative stress

(A) Top enriched pathways using gene set enrichment analysis (GSEA) of RNA-seq dataset comparing hearts from 3-month-old saline or doxorubicin (DOXO; 48 h 25mg/kg) treated control and iSOD2 female mice. FDR, false discovery rate (n=3-4 mice per condition). **(B)** GSEA of pathways pertaining to heart development from RNA-seq dataset illustrating a significant underrepresentation of fetal cardiac gene expression in DOXO treated iSOD2 mice vs. DOXO treated controls (n=3-4 mice per condition). **(C)** Heatmap of the top 500 genes with differential expression following DOXO treatment in controls and illustrating a dampening of the DOXO-induced transcriptional response in iSOD2 mice. Color depicts z-score (n=3-4 mice per condition). **(D)** Heatmap depicting the change in expression of genes in Mitocarta3.0 database following DOXO treatment in each genotype relative to its saline-treated control. Color depicts log2FoldChange (n=3-4 mice per condition). **(E)** Significant Mitocarta-specific GSEA hits between DOXO-treated control and iSOD2 mice (n=3-4 mice per condition). **(F)** Densitometry analysis of OXPHOS proteins from western blot analysis in Figure 2G of vehicle or DOXO treated control and iSOD2 mice (n=3-4 mice per condition). **(G)** RT-qPCR examining *Sod2* expression from cardiac lysate in response to acute DOXO treatment (n=5-6 mice per condition). **(H)** Densitometry of SOD2 protein expression from Figure 2H in acute DOXO treated control and iSOD2 hearts (n=5-6 mice per condition). Statistical significance is determined using two-way ANOVA with Šidák multiple comparison test correction. *p<0.01, ****p<0.0001, ns is not significant. Histograms represent mean ± SEM.

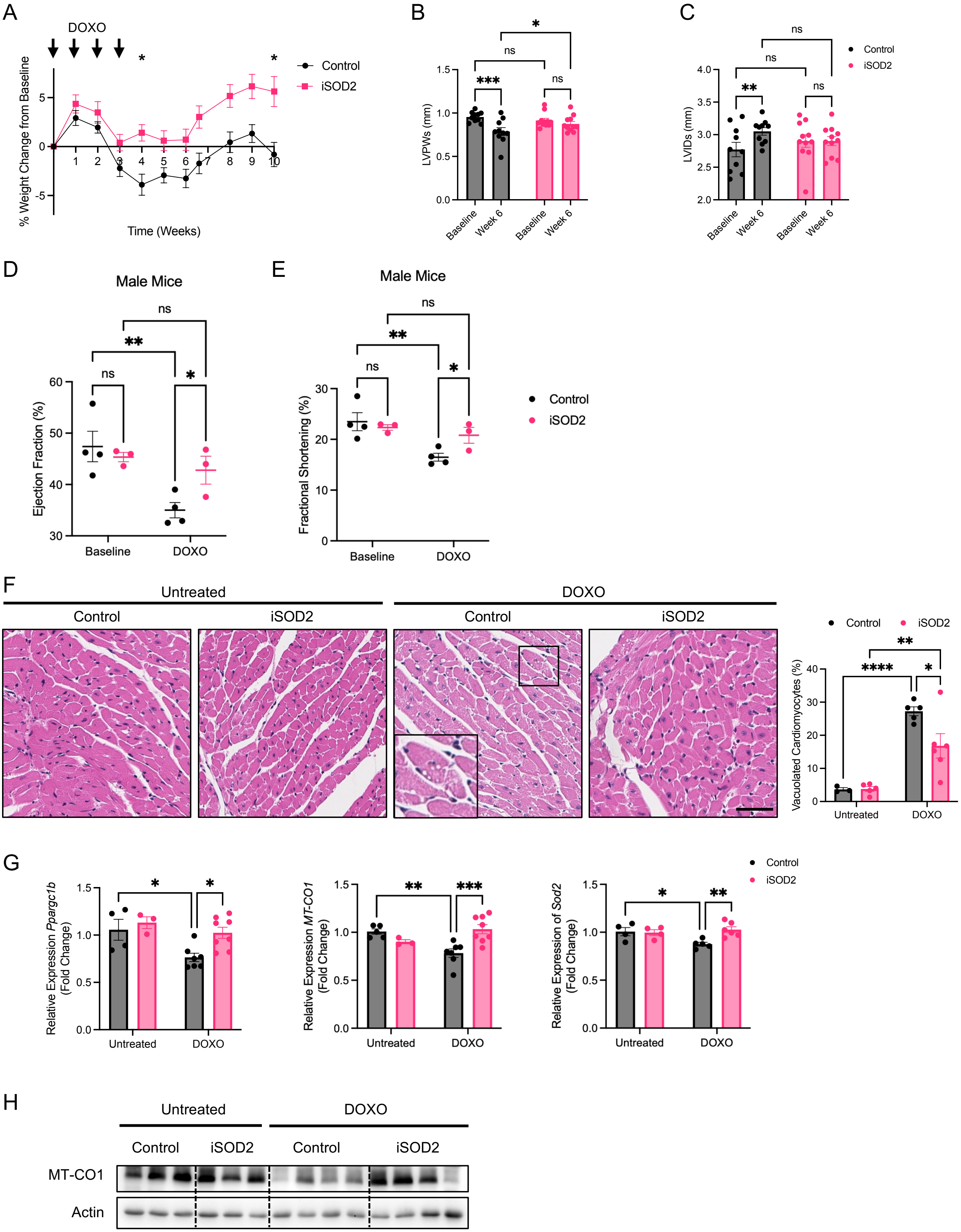
A
DOXO
% Weight Change from Baseline
Time (Weeks)
Control
iSOD2
B
LVPWs (mm)
Baseline
Week 6
C
LVIDs (mm)
D
Male Mice
Ejection Fraction (%)
DOXO
E
Male Mice
Fractional Shortening (%)
F
Untreated
Control
iSOD2
DOXO
Vacuolated Cardiomyocytes (%)
G
Relative Expression Ppargc1b (Fold Change)
Relative Expression MT-CO1 (Fold Change)
Relative Expression of Sod2 (Fold Change)
H
MT-CO1
Actin

Fig. S3. Matrix superoxide-mediated mitohormesis dampens signatures of chronic doxorubicin-induced cardiotoxicity

(A) Weekly percent weight change from baseline over the course of the chronic doxorubicin (DOXO) injury model in 3-month-old control and iSOD2 mice (n=10-11 mice per condition). **(B)** Left ventricular posterior wall thickness end-systole ($LVPW_s$) in control and iSOD2 mice 6-weeks following the initial DOXO injection relative to their baseline (n=10-11 mice per condition). **(C)** Left ventricular internal diameter end-systole ($LVID_s$) in control and iSOD2 mice 6-weeks post initial DOXO injection relative to their baseline (n=10-11 mice per condition). **(D)** Ejection fraction and **(E)** fractional shortening of control (n=4) and iSOD2 (n=3) male mice comparing baseline function to 6-weeks following first DOXO injection. **(F)** Representative H&E images depicting cardiomyocyte vacuolization following DOXO treatment in either 5.5-month-old untreated or DOXO-treated control and iSOD2 mice. (10 weeks following initial DOXO injection). Quantification depicts percent of cardiomyocytes with one vacuole from 6 different fields of view per mouse (n=3-6 mice per condition). Scale bar is 50 μm. **(G)** RT-qPCR analysis of *Ppargc1b*, *MT-Co1*, and *Sod2* (normalized to *Hmbs*) expression from heart lysates of untreated or DOXO treated control and iSOD2 mice (n=3-6 mice per condition). **(H)** Immunoblot depicting expression of MT-CO1 from heart lysates of untreated or DOXO-treated control and iSOD2 mice (n=3-6 mice per condition). Statistical significance determined using two-way ANOVA with Šidák multiple comparison test correction. *$p<0.05$, **$p<0.01$, ***$p<0.001$, ****$p<0.0001$, ns is not significant. Histograms represent mean ± SEM.

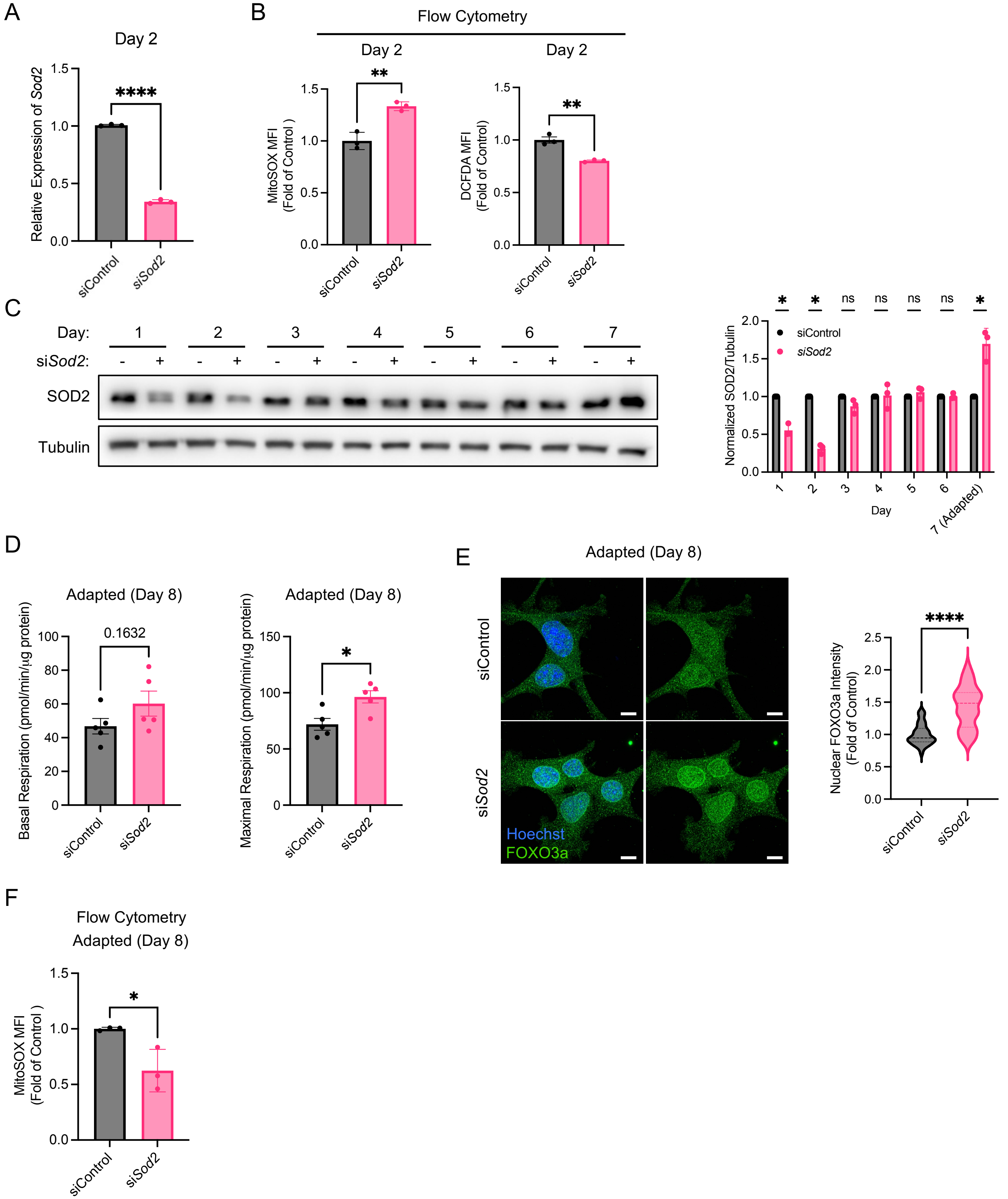

A
Day 2
Relative Expression of Sod2
****
siControl
siSod2
B
Flow Cytometry
Day 2
MitoSOX MFI (Fold of Control)
**
DCFDA MFI (Fold of Control)
C
Day:
siSod2:
SOD2
Tubulin
Normalized SOD2/Tubulin
siControl
siSod2
ns
Day
7 (Adapted)
D
Adapted (Day 8)
Basal Respiration (pmol/min/μg protein)
0.1632
Maximal Respiration (pmol/min/μg protein)
E
Adapted (Day 8)
Hoechst
FOXO3a
Nuclear FOXO3a Intensity (Fold of Control)
F
Flow Cytometry
Adapted (Day 8)
MitoSOX MFI (Fold of Control)

fig. S4

Fig. S4. Transient *Sod2* silencing in mouse embryonic fibroblasts phenocopies matrix superoxide-mediated mitohormetic effects from hearts of iSOD2 mice

(A) RT-qPCR analysis depicting *Sod2* depletion 48 h following siRNA transfection in mouse embryonic fibroblasts (MEFs). Graph depicts technical triplicates from one experiment representative of 3 biological replicates. **(B)** Flow cytometry analysis 48 h following *Sod2* knockdown measuring mitochondrial ROS and cellular ROS using MitoSOX and DCFDA, respectively. **(C)** Representative immunoblot and densitometry depicting SOD2 re-expression kinetics over a 7-day time course following *Sod2* knockdown. **(D)** Quantification of mitochondrial respiration using Seahorse analysis from Figure 4G. **(E)** Representative immunofluorescence imaging and quantification illustrating sustained FOXO3a nuclear localization in adapted MEFs 8 days following *Sod2* knockdown. Nuclear FOXO3a is quantified by masking the nuclear signal with Hoechst and calculating the integrated density of FOXO3a signal (n=40 cells per condition). **(F)** Flow cytometry analysis of adapted (Day 8) SOD2 MEFs measuring mitochondrial ROS with MitoSOX. All experiments were conducted with a minimum of n=3 biological replicates. Statistical significance is determined using unpaired Student's *t*-test. *$p<0.05$, **$p<0.01$, ****$p<0.0001$, ns is not significant. Histograms represent mean ± SEM.

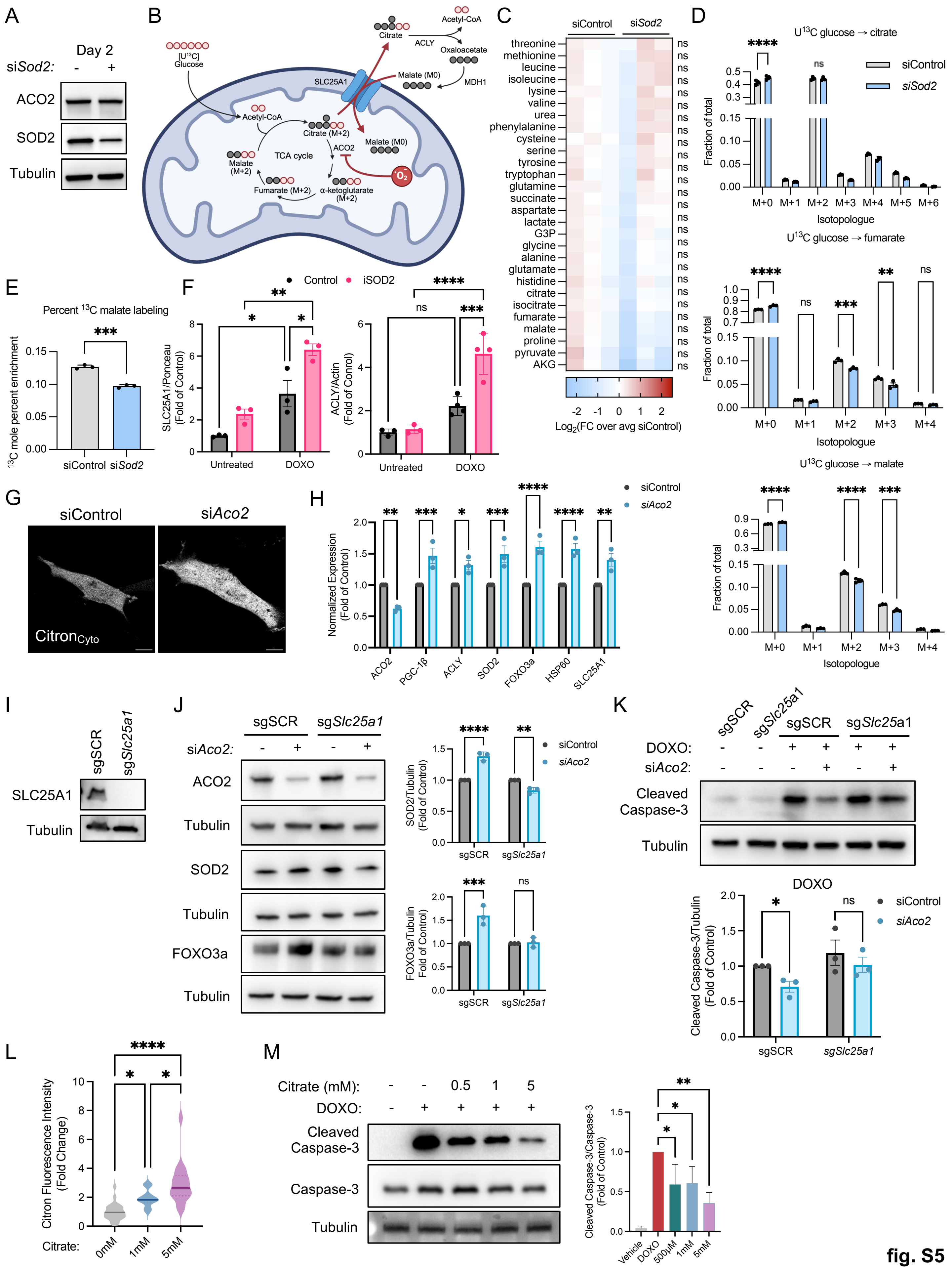

A
Day 2
siSod2: - +
ACO2
SOD2
Tubulin
B
[U13C] Glucose
Acetyl-CoA
Citrate
ACLY
Oxaloacetate
MDH1
Malate (M0)
SLC25A1
Citrate (M+2)
Malate (M0)
ACO2
TCA cycle
Malate (M+2)
Fumarate (M+2)
α-ketoglutarate (M+2)
C
siControl
siSod2
threonine
methionine
leucine
isoleucine
lysine
valine
urea
phenylalanine
cysteine
serine
tyrosine
tryptophan
glutamine
succinate
aspartate
lactate
G3P
glycine
alanine
glutamate
histidine
citrate
isocitrate
fumarate
malate
proline
pyruvate
AKG
Log2(FC over avg siControl)
D
U13C glucose → citrate
U13C glucose → fumarate
U13C glucose → malate
Fraction of total
Isotopologue
siControl
siSod2
E
Percent 13C malate labeling
13C mole percent enrichment
siControl siSod2
F
Control iSOD2
SLC25A1/Ponceau (Fold of Control)
ACLY/Actin (Fold of Control)
Untreated DOXO
G
siControl
siAco2
CitronCyto
H
siControl
siAco2
Normalized Expression (Fold of Control)
ACO2 PGC-1β ACLY SOD2 FOXO3a HSP60 SLC25A1
I
sgSCR sgSlc25a1
SLC25A1
Tubulin
J
sgSCR sgSlc25a1
siAco2: - + - +
ACO2
Tubulin
SOD2
Tubulin
FOXO3a
Tubulin
SOD2/Tubulin (Fold of Control)
FOXO3a/Tubulin Fold of Control)
K
sgSCR sgSlc25a1 sgSCR sgSlc25a1
DOXO: - - + + + +
siAco2: - - - + - +
Cleaved Caspase-3
Tubulin
DOXO
Cleaved Caspase-3/Tubulin (Fold of Control)
L
Citron Fluorescence Intensity (Fold Change)
Citrate: 0mM 1mM 5mM
M
Citrate (mM): - - 0.5 1 5
DOXO: - + + + +
Cleaved Caspase-3
Caspase-3
Tubulin
Cleaved Caspase-3/Caspase-3 (Fold of Control)
Vehicle DOXO 500μM 1mM 5mM
fig. S5

Fig. S5. Mitochondrial citrate signaling is required for matrix superoxide-induced mitohormetic adaptations

(A) Immunoblot analysis illustrating SOD2 loss and no change in ACO2 expression in MEFs used for aconitase rate from Figure 5A. **(B)** Schematic depicting tracing experiment with [U-^{13}C] glucose (red). Map illustrates a superoxide mediated inhibition of aconitase, thereby re-routing citrate for cytosolic processing by ACLY. Aconitase (ACO2), superoxide (O_2^-), mitochondrial citrate transporter (SLC25A1), ATP citrate lyase (ACLY). **(C)** Heatmap depicting normalized abundance of tricarboxylic acid (TCA) cycle metabolites, amino acids, and glycolytic intermediates 48 h following *Sod2* knockdown in MEFs. **(D)** Mass isotopologue distributions of TCA cycle intermediates following 1 h incubation with [U-^{13}C] glucose, 48 h following *Sod2* knockdown. **(E)** Mole percent enrichment of ^{13}C labeled malate between negative control and si*Sod2* MEFs at 48 h post transfection following 1 h of labeling. **(F)** Densitometry analysis of SLC25A1 and ACLY illustrating a preferential increase in both proteins in hearts from iSOD2 mice following chronic DOXO treatment from Figure 5E. **(G)** Representative images showing cytosolic citrate fluorescence 48 h following *Aco2* knockdown from Fig. 5f. **(H)** Densitometry analysis from immunoblots in Figure 5G of mitohormetic signature following 48 h of *Aco2* knockdown. **(I)** Western blot confirmation of SLC25A1 knockout sg*Slc25a1* MEFs relative to scramble (sgSCR) control MEFs. **(J)** Representative immunoblot and densitometry analysis illustrating the ACO2-mediated increase in SOD2 and FOXO3a 48 h following si*Aco2* transfection is SLC25A1 dependent. **(K)** Representative immunoblot illustrating the ACO2-mediated protection from DOXO (100 nM for 24 h) is SLC25A1 dependent. **(L)** Quantification of increase in $Citron_{cyto}$ fluorescence following incubation with increasing concentrations of exogenous citrate for 24 h. **(M)** Representative immunoblot and quantification illustrating protection from doxorubicin (DOXO; 100 nM for 24 h) cell death following 24 h pre-treatment with increasing concentrations of exogenous citrate. All experiments were conducted with a minimum of 3 biological replicates, except A which was conducted in duplicate. Statistical significance is determined using unpaired Student's *t*-test for (C-H), two-way ANOVA with Šidák multiple comparison test correction for (J and K), and one-way ANOVA with Dunnett's multiple comparisons test for (L and M). *$p<0.05$, **$p<0.01$, ***$p<0.001$, ****$p<0.0001$, ns is not significant. Histograms represent mean ± SEM. Schematic created in BioRender. Donnelly, M. (2026) https://BioRender.com/eh0fznl.

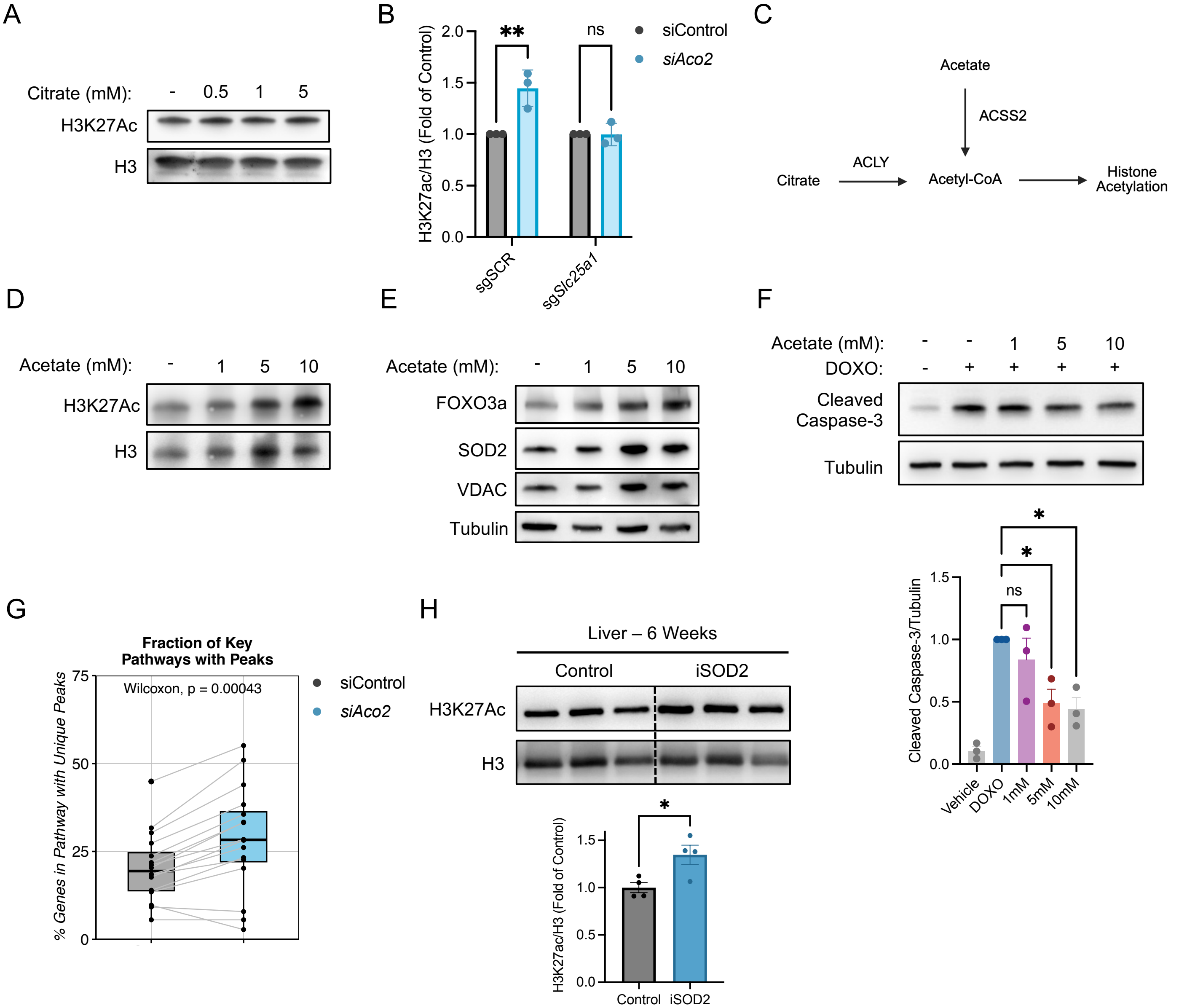

A
Citrate (mM): - 0.5 1 5
H3K27Ac
H3
B
H3K27ac/H3 (Fold of Control)
2.0
1.5
1.0
0.5
0.0
**
ns
siControl
siAco2
sgSCR
sgSlc25a1
C
Acetate
ACSS2
Citrate
ACLY
Acetyl-CoA
Histone Acetylation
D
Acetate (mM): - 1 5 10
H3K27Ac
H3
E
Acetate (mM): - 1 5 10
FOXO3a
SOD2
VDAC
Tubulin
F
Acetate (mM): - - 1 5 10
DOXO: - + + + +
Cleaved Caspase-3
Tubulin
Cleaved Caspase-3/Tubulin
1.5
1.0
0.5
0.0
*
*
ns
Vehicle
DOXO
1mM
5mM
10mM
G
Fraction of Key Pathways with Peaks
Wilcoxon, p = 0.00043
% Genes in Pathway with Unique Peaks
75
50
25
0
siControl
siAco2
H
Liver – 6 Weeks
Control
iSOD2
H3K27Ac
H3
H3K27ac/H3 (Fold of Control)
1.5
1.0
0.5
0.0
*
Control
iSOD2

fig. S6

Fig. S6. Matrix superoxide-induced mitohormesis increases histone acetylation via mitochondrial citrate-derived acetyl-CoA

(A) Representative immunoblot analysis depicting a dose-dependent increase in histone acetylation following 24 h treatment with exogenous citrate in MEFs. **(B)** Densitometry analysis of representative immunoblots from Figure 6E illustrating the increase in H3K27Ac driven by si*Aco2* is SLC25A1 dependent. **(C)** Schematic illustrating routes of acetyl-CoA generation in the cytosol from citrate via ACLY and acetate via ACSS2. **(D)** Representative western blot analysis of MEFs treated with increasing concentrations of exogenous acetate for 24 h illustrating an increase in H3K27Ac. **(E)** Representative immunoblot analysis depicting an increase in mitohormesis markers FOXO3a, SOD2, and VDAC with pre-treatment of high dose acetate for 24 h. **(F)** Representative immunoblot and densitometry depicting protection from doxorubicin (DOXO)-induced cell death with increasing doses of acetate pre-treatment for 24 h. **(G)** Quantification of the percent of genes gaining unique H3K27Ac peaks following 48 h of *Aco2* knockdown. Each data point represents one gene set depicted in Fig. 6I. **(H)** Representative western blot analysis of increased H3K27Ac in livers isolated from 6-week-old littermate control and iSOD2 mice (n=4 mice). All cell culture experiments were conducted in 3 biological replicates, except for (A) which was conducted in biological duplicate. Statistical significance was determined using two-way ANOVA with Šidák multiple comparison test correction for (B), one-way ANOVA with Dunnett's multiple comparisons test for (F), paired Wilcoxin test for (G), and unpaired Student's *t*-test for (H). *$p<0.05$, **$p<0.01$. Histograms represent mean ± SEM.

Table S1. Significant differentially expressed genes ($p < 0.05$) in cardiac lysates isolated from littermate control and iSOD2 mice, related to Figure 1.

List of significant differentially expressed genes from bulk RNA-sequencing of hearts isolated from littermate control and iSOD2 mice at 6 weeks. Differences between groups are represented as log2FoldChange and significant genes have and adjusted p-value of <0.05. n=5 mice per condition.

Table S2. Normalized counts from RNA-seq of saline or doxorubicin challenged control and iSOD2 mice, related to Figure 2.

Excel datasheet containing normalized counts from bulk RNA-sequencing dataset of hearts from 3-month-old control and iSOD2 mice 48 h after receiving either saline or doxorubicin (25mg/kg). (n=3-4 mice per condition).

Table S3. Oligonucleotide sequences of siRNAs, primers, and gRNAs

Oligonucleotide sequences of siRNAs, shRNAs, primers, and gRNAs used throughout the manuscript.
